# The novel adrenergic agonist ATR-127 targets skeletal muscle and brown adipose tissue to tackle diabesity and steatohepatitis

**DOI:** 10.1016/j.molmet.2024.101931

**Published:** 2024-05-17

**Authors:** Emanuela Talamonti, Jelena Davegardh, Anastasia Kalinovich, Sten M.M. van Beek, Nodi Dehvari, Carina Halleskog, Hamza M. Bokhari, Dana S. Hutchinson, Seungmin Ham, Laura J. Humphrys, Nicola C. Dijon, Aikaterini Motso, Anna Sandstrom, Evelyn Zacharewicz, Ilga Mutule, Edgars Suna, Jana Spura, Karolina Ditrychova, Leigh A. Stoddart, Nicholas D. Holliday, Shane C. Wright, Volker M. Lauschke, Soren Nielsen, Camilla Scheele, Elizabeth Cheesman, Joris Hoeks, Peter Molenaar, Roger J. Summers, Benjamin Pelcman, Gopala K. Yakala, Tore Bengtsson

**Affiliations:** 1 Atrogi AB, Tomtebodavagen 6, Solna, Stockholm, Sweden; 2 Department of Molecular Biosciences, The Wenner-Gren Institute, Stockholm University, Stockholm, Sweden; 3 Drug Discovery Biology, Monash Institute of Pharmaceutical Sciences, Monash University, Parkville, Victoria, Australia; 4 School of Life Sciences, The Medical School, Queen's Medical Centre, University of Nottingham, Nottingham, UK; 5 Karolinska Institutet, Department of Physiology and Pharmacology, Stockholm, Sweden; 6 Department of Nutrition and Movement Sciences, NUTRIM School of Nutrition and Translational Research in Metabolism, Maastricht University Medical Center, Maastricht, the Netherlands; 7 Latvian Institute of Organic Synthesis, Riga, Latvia; 8 Excellerate Bioscience, The Triangle, NG2 Business Park, Nottingham, UK; 9 Dr. Margarete Fischer-Bosch Institute of Clinical Pharmacology, Stuttgart, Germany; 10 Tübingen University, Tübingen, Germany; 11 Novo Nordisk Foundation Center for Basic Metabolic Research, Faculty of Health and Medical Sciences, University of Copenhagen, Copenhagen, Denmark; 12 The Centre of Inflammation and Metabolism and Centre for Physical Activity Research, Righospitalet, University Hospital of Copenhagen, Copenhagen, Denmark; 13 Cardio-Vascular Molecular & Therapeutics Translational Research Group, Northside Clinical School of Medicine, Faculty of Medicine, University of Queensland, Brisbane, Queensland, Australia; 14 Queensland University of Technology (QUT), School of Biomedical Sciences, Institute of Health and Biomedical Innovation, 60 Musk Avenue, Kelvin Grove, Queensland, Australia

**Keywords:** Obesity, Type 2 diabetes, β-Adrenergic agonists, Hepatic steatosis, Skeletal muscle

## Abstract

**Objective:**

Simultaneous activation of β2- and β3-adrenoceptors (ARs) improves whole-body metabolism via beneficial effects in skeletal muscle and brown adipose tissue (BAT). Nevertheless, high-efficacy agonists simultaneously targeting these receptors whilst limiting activation of β1-ARs – and thus inducing cardiovascular complications – are currently non-existent. Therefore, we here developed and evaluated the therapeutic potential of a novel β2-and β3-AR, named ATR-127, for the treatment of obesity and its associated metabolic perturbations in preclinical models.

**Methods:**

In the developmental phase, we assessed the impact of ATR-127's on cAMP accumulation in relation to the non-selective β-AR agonist isoprenaline across various rodent β-AR subtypes, including neonatal rat cardiomyocytes. Following these experiments, L6 muscle cells were stimulated with ATR-127 to assess the impact on GLUT4-mediated glucose uptake and intramyocellular cAMP accumulation. Additionally, *in vitro*, and *in vivo* assessments are conducted to measure ATR-127's effects on BAT glucose uptake and thermogenesis. Finally, diet-induced obese mice were treated with 5 mg/kg ATR-127 for 21 days to investigate the effects on glucose homeostasis, body weight, fat mass, skeletal muscle glucose uptake, BAT thermogenesis and hepatic steatosis.

**Results:**

Exposure of L6 muscle cells to ATR-127 robustly enhanced GLUT4-mediated glucose uptake despite low intramyocellular cAMP accumulation. Similarly, ATR-127 markedly increased BAT glucose uptake and thermogenesis both *in vitro* and *in vivo*. Prolonged treatment of diet-induced obese mice with ATR-127 dramatically improved glucose homeostasis, an effect accompanied by decreases in body weight and fat mass. These effects were paralleled by an enhanced skeletal muscle glucose uptake, BAT thermogenesis, and improvements in hepatic steatosis.

**Conclusions:**

Our results demonstrate that ATR-127 is a highly effective, novel β2- and β3-ARs agonist holding great therapeutic promise for the treatment of obesity and its comorbidities, whilst potentially limiting cardiovascular complications. As such, the therapeutic effects of ATR-127 should be investigated in more detail in clinical studies.

## Highlights

• ATR-127 a safe dual β2-β3-AR agonist, demonstrates improved cardiovascular safety.
• ATR-127 enhances skeletal muscle glucose uptake independently of cAMP.
• ATR-127 markedly increases BAT glucose uptake and thermogenesis.
• ATR-127 improves hepatic steatosis.
• Prolonged ATR-127 treatment exhibits potent antiobesity and antidiabetic effects.

## 1 Introduction

Due to its rapidly growing prevalence, obesity has been declared as an epidemic by the World Health Organization [1]. Obesity is a serious, chronic metabolic disorder leading to excessive lipid storage in non-adipose tissues [2]. This ectopic fat accumulation is highly associated with the development of severe comorbidities such as non-alcoholic fatty liver disease and type 2 diabetes mellitus (T2DM) [2,3]. Current treatment options for obesity and its comorbidities are limited to lifestyle interventions that are difficult to comply with [[4], [5], [6]] or bariatric surgery, which is only recommended for the most severe cases due to associated complications [7]. Although there exist limited pharmacological options, they come with notable constraints [8]. For example, GLP-1 agonists have been shown to reduce skeletal muscle mass, raising concerns about their potential implications for various comorbidities in the future [9,10]. Additionally, the use of GLP-1 agonists has been associated with an increased risk of pancreatitis, gastroparesis, and bowel obstruction in patients [11]. Therefore, even with substantial research conducted in this field, there is an immediate need for innovative pharmacological strategies.

As obesity originates from excessive lipid storage, pharmacological compounds that enhance energy expenditure and substrate utilization are promising in preventing excessive calorie storage and improving metabolic health. In this context, recent studies have focused on the potential of targeting the β-adrenergic receptor (β-AR) to combat obesity and its associated metabolic disturbances. β-ARs are widely expressed in tissues [12] that are known to drastically affect energy expenditure and substrate oxidation in humans [13,14], rendering them attractive targets for anti-obesity medication. However, currently used β-AR agonists are frequently associated with increases in heart rate and blood pressure [15]. As such, further characterisation of metabolic tissues and the associated β-AR subtypes mediating beneficial effects, as well as understanding the underlying mechanisms of side effects, could lead to the development of a novel class of β-AR agonists to combat obesity.

Skeletal muscle is a highly active metabolic organ identified as an important source of the thermogenic effects of β-AR agonists. These effects result almost exclusively from β_2_-AR activation [[16], [17], [18], [19]]. Furthermore, treatment with a selective β_2_-AR agonist has been reported to enhance energy expenditure and substrate oxidation in humans [17,20,21]. Some studies have even reported a significant reduction in fat mass in humans upon β_2_-AR agonist treatment [22,23]. Besides these effects on energy expenditure, we have previously described a novel pathway mediating glucose uptake in skeletal muscle following β_2_-AR activation [24]. This resulted in robustly enhanced skeletal muscle glucose uptake *in vivo* in mice [25] and markedly improved glucose homeostasis in insulin resistant rodents [[24], [25], [26], [27], [28], [29], [30]]. This therefore strongly suggests that the β_2_-AR is an interesting target to treat obesity and diabetes, also referred to as ‘diabesity’.

Besides skeletal muscle, brown adipose tissue (BAT) has the capacity to increase thermogenesis and substrate oxidation upon β-AR stimulation and is an attractive target tissue for the treatment of obesity [31]. Indeed, stimulation of β_3_-AR, the main receptor implicated in murine BAT activation [32], increases energy expenditure and substrate oxidation [33,34], reduces body weight and fat mass [35], and even improves glucose homeostasis in rodent models of diabetes [36,37]. Humans retain BAT in adulthood [[38], [39], [40]], that appears to be activated by β_2_-AR [41,42] or β_3_-AR [43]. These findings therefore suggest that similar beneficial effects on body composition and glucose homeostasis could be achieved by activation of human BAT with β_2_ or β_3_-AR agonists.

Combined, these results suggest that activation of both subtypes with a β_2_-β_3_-AR dual agonist would effectively combat obesity and its related metabolic perturbations, while largely limiting cardiovascular side-effects by avoiding β_1_-AR stimulation. Based on this knowledge, we have developed a novel β-adrenergic agonist, ATR-127, and here we present its metabolic effects. Specifically, we have analysed responses *in vitro* in skeletal muscle and BAT cells, as well as *in vivo* in diet-induced obese (DIO) mice. In short, we demonstrate that ATR-127 increases skeletal muscle and BAT glucose uptake and increases BAT and whole-body thermogenesis. Chronic ATR-127 treatment markedly improves glucose homeostasis and reduces body weight, fat mass, and hepatic steatosis in DIO mice.

## 2 Results

### 2.1 Differential potency of ATR-127 in cAMP accumulation via β-ARs compared to isoprenaline

**Figure 1:**
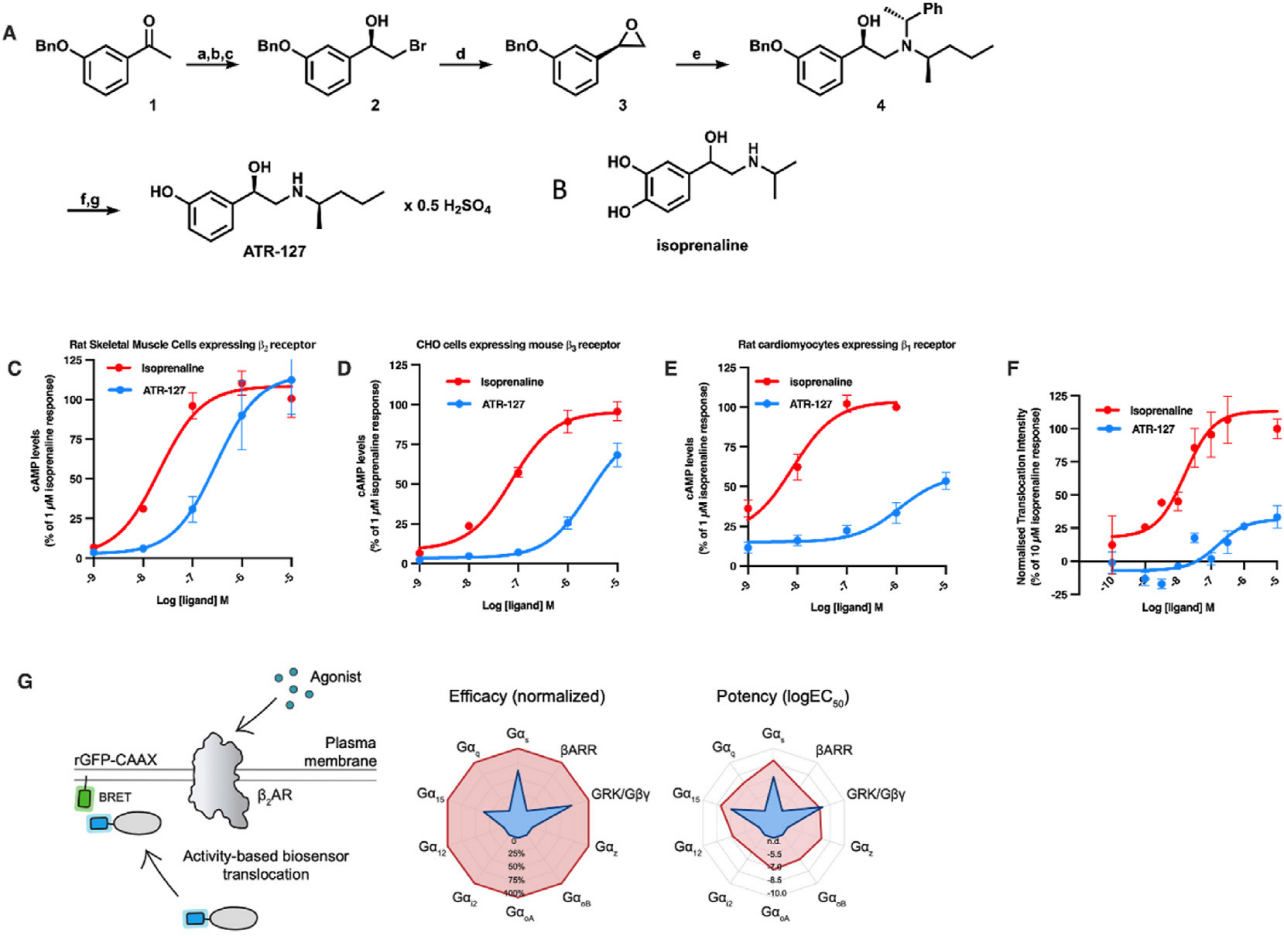
**ATR-127, a novel adrenergic agonist, exhibits differential cAMP generation capability and minimal desensitization response.** (A) The synthesis of 3-((*R*)-1-hydroxy-2-(((*R*)-pentan-2-yl)amino)ethyl)phenol (ATR-127). (a) Br_2_, CHCl_3_, reflux, 5 h; (b) diethyl phosphite, Et_3_N, THF, 0 °C to rt, 1 h (63% over two steps); (c) BH_3_·Me_2_S, (*R*)-2-methyl-CBS-oxaborolidine, PhMe, THF, 0 °C to rt, 2 h (91%, 98% ee); (d) K_2_CO_3_, MeOH, rt, 1 h (95%); (e) (*R*)-*N*-((*R*)-1-phenylethyl)pentan-2-amine, *i*-PrOH, 140 °C (sealed tube), 88 h (72% based on the amine); (f) Pd-C (10%), Et_3_SiH, MeOH, rt, 1 h (74%); (g) 0.5 eq H_2_SO_4_, H_2_O, rt, 1 h (82%). (B) Chemical structure of isoprenaline. (C–E) cAMP concentration response curves upon stimulation with ATR-127 or isoprenaline in cells expressing rodent adrenergic β receptors, (C) Rat skeletal muscle cell line expressing β_2_AR. (D) CHO cells expressing mouse β_3_AR. (E) Rat cardiomycytes expressing β_1_AR. (F) β_2_AR translocation intensity normalized to isoprenaline. (G) Characterization of transducer engagement at the β_2_AR by ebBRET. As depicted in the illustration, donor-tagged, pathway-selective biosensors are co-expressed with membrane-anchored acceptor and the receptor is stimulated with agonist (isoprenaline or ATR-127) to monitor pathway activation by measuring BRET. Radar plots are shown for efficacy (normalized to isoprenaline) and potency (logEC_50_) of the pathways engaged by the β_2_AR at the plasma membrane. Drugs were deemed to activate a given pathway after comparing the top and bottom parameters from non-linear regression by one-sided extra sum-of-squares F test followed by the Benjamini-Hochberg correction (*P* < 0.0043).

As ATR-127 is a newly developed β-AR agonist (Figure 1A), with a similar chemical structure as isoprenaline (Figure 1B), we conducted an assessment of ATR-127's impact on cAMP accumulation in relation to the non-selective β-AR agonist isoprenaline across various rodent β-AR subtypes (Figure 1C–E). ATR-127 demonstrated the characteristics of a full agonist but with a reduced potency compared to isoprenaline in both L6 cells with endogenous expression of the rat β_2_-AR (isoprenaline logEC50–7.6 and ATR-127 logEC50–6.53) (Figure 1C) and CHO cells stably expressing mouse β_3_-AR (isoprenaline logEC50–7.1 and ATR-127 logEC50–5.5) (Figure 1D). In contrast, when assessed against the rat β_1_-AR, which is naturally expressed in neonatal rat cardiomyocytes, ATR-127 displayed weaker partial agonist activity compared to isoprenaline (isoprenaline logEC50–8.0 and ATR-127 logEC50–5.95) (Figure 1E).

In addition, agonist-induced internalisation of the β_2_-AR (i.e., the desensitization response) was significantly lower, with a maximal efficacy of only 28% of isoprenaline with 10-fold lower potency (logEC50–6.7) than isoprenaline (logEC50–7.7) (Figure 1F).

To further investigate transducer engagement by the β_2_-AR, we performed enhanced bystander bioluminescence resonance energy transfer (ebBRET) to monitor agonist-induced signaling at the plasma membrane. Stimulation with ATR-127 led to the selective engagement of G_s_, G_15_, and GRK/Gβγ signaling pathways, whereas the addition of isoprenaline led to more extensive transducer coupling to β_2_-AR in line with previous observations [44] (Figure 1G, Suppl figure 1).

### 2.2 ATR-127 increases glucose uptake in skeletal muscle by stimulating β_2_-adrenoceptors

**Figure 2:**
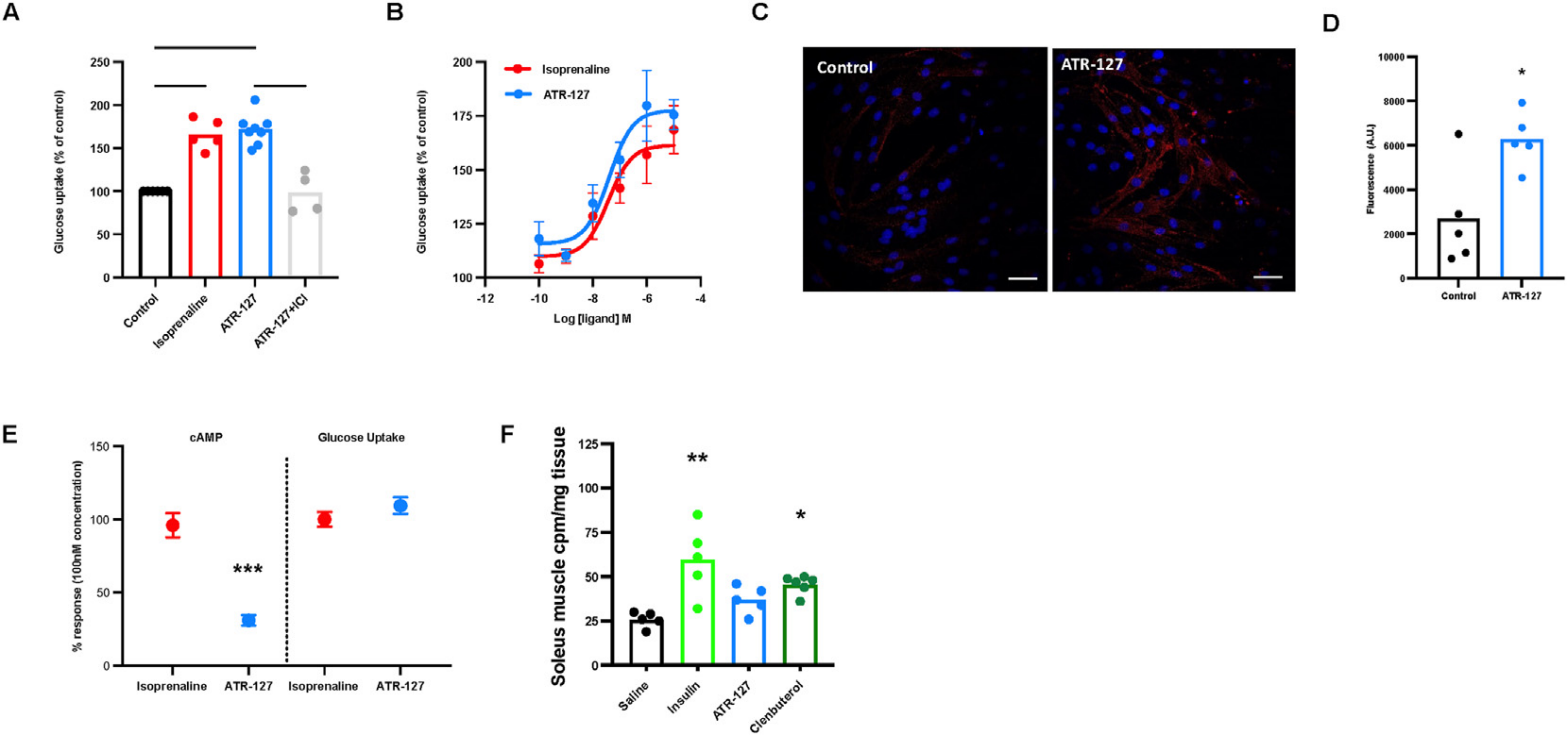
**ATR-127 robustly increases skeletal muscle glucose uptake via GLUT4 translocation.** (A) Glucose uptake in L6 cells upon isoprenaline (1 μM), ATR-127 (1 μM) or ATR-127 + ICI-118,551 (1 μM) incubation. (B) Dose-response curves of glucose uptake in L6 cells treated with isoprenaline and ATR-127. (C) Representative fluorescent images of Glut-4 translocation to the membranes of L6 cells upon treatment with ATR-127 (10 μM) (Red: Glut-4 protein, blue: nucleus) with scale bars (50 μm). (D) Quantification of Glut-4 translocation to the plasma membrane. (E) cAMP levels (from Figure 1C) and glucose uptake levels (from Figure 2B) at 100 nM concentration (F) *In vivo* glucose uptake in soleus muscle upon acute injection with saline, insulin (1 mg/kg), ATR-127 (1 mg/kg) or clenbuterol (1 mg/kg). One data point was removed in Figure 2F based on outlier test. In the event of normal distribution, data were analyzed by means of the Student's *t* test or one-way ANOVA followed by the Dunnett's or Sidak's multiple comparison tests. ∗p < 0.05, ∗∗∗p < 0.001, ∗∗∗∗p < 0.0001. (For interpretation of the references to color in this figure legend, the reader is referred to the Web version of this article.)

Next, we investigated the effects of ATR-127 on *in vitro* and *in vivo* skeletal muscle glucose uptake. In L6-Glut4myc myotubes, both the non-selective β-AR agonist isoprenaline (10 μM) and ATR-127 (10 μM) increased glucose uptake to the same extent and in a concentration-dependent manner (Figure 2A–B). The effects of ATR-127 on glucose uptake were completely abolished by prior incubation with the selective β_2_-AR antagonist ICI-118,551 (10 μM) (Figure 2A), confirming that ATR-127 exerts its effects on glucose uptake via the β_2_-AR. The glucose uptake by skeletal muscle cells following stimulation with ATR-127 (10 μM) was associated with a significant increase in GLUT4 translocation (p ≤ 0.01) (Figure 2C–D). Interestingly, for cells treated with ATR-127 the increase in glucose uptake occurred despite markedly reduced intracellular cAMP accumulation at 100 nM concentration (p ≤ 0.0001) (Figure 2E; cAMP levels of 100 nM concentration from Rat skeletal muscle L6-cells, derived from Figure 1C and glucose uptake levels of 100 nM concentration from Rat skeletal muscle L6-cells, derived from Figure 2B). To investigate whether similar effects are observed *in vivo,* C57Bl/N6 mice were injected with saline, insulin (1 mg/kg), ATR-127 (1 mg/kg) or the selective β_2_-AR agonist clenbuterol (1 mg/kg) and skeletal muscle glucose uptake was measured. Both insulin and clenbuterol increased skeletal muscle glucose uptake (Figure 2F) and a tendency to increased glucose uptake was observed for ATR-127, though did not reach statistical significance, probably due to lack of power.

### 2.3 ATR-127 induces glucose uptake and thermogenesis in brown adipocytes

**Figure 3:**
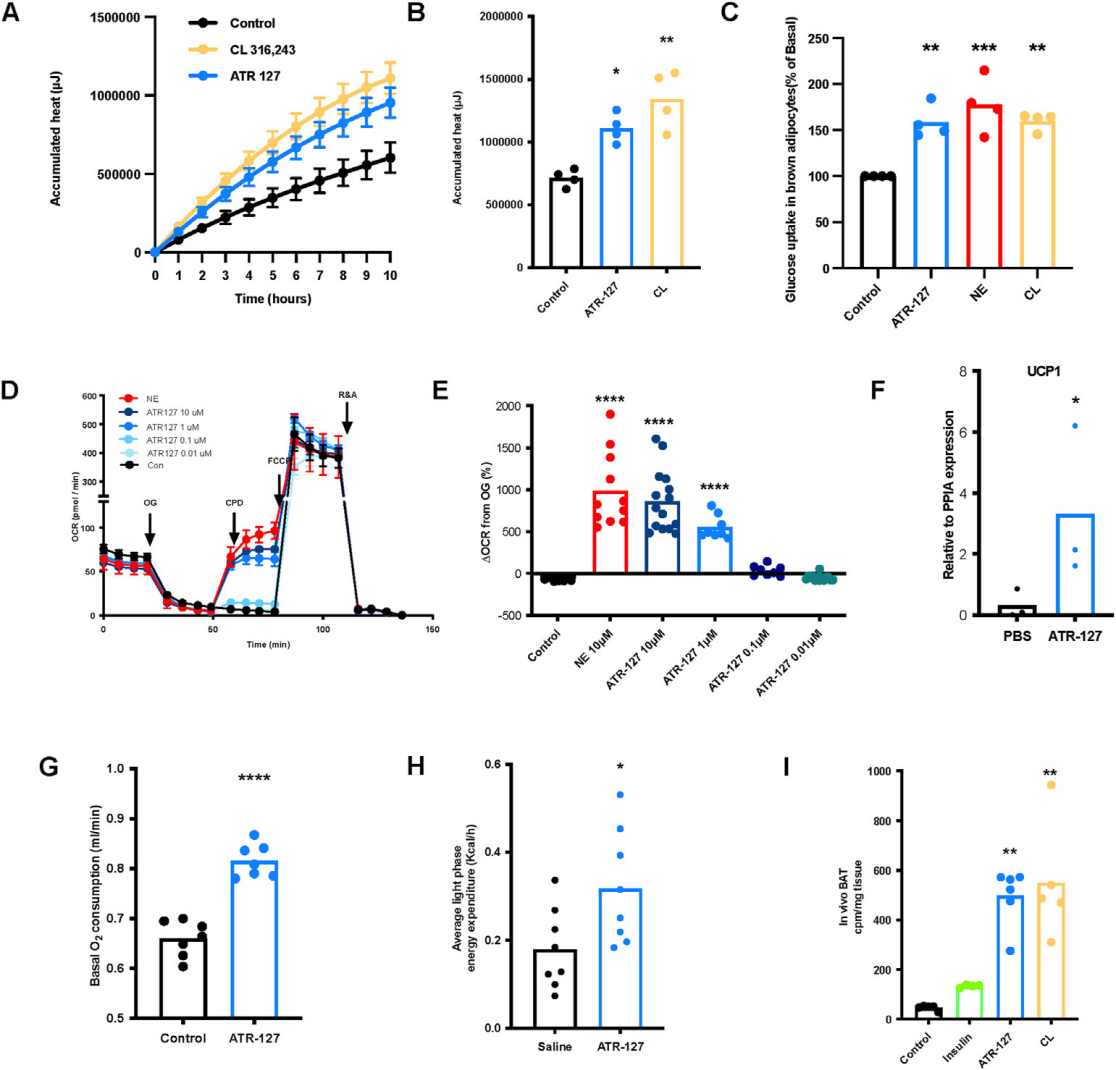
**Treatment with ATR-127 enhances *in vitro* and *in vivo* BAT thermogenesis and glucose uptake** (A) Accumulated heat production over time of brite adipocytes stimulated with saline, CL-316,243 (1 μM) or ATR-127 (1 μM). (B) Total accumulated heat production of brite adipocytes stimulated with saline, CL-316,243 or ATR-127. (C) Glucose uptake in *ex vivo* brown adipose tissue upon stimulation with ATR-127 (1 μM), NE (1 μM) and CL-316,243 (1 μM). (D) Oxygen consumption rate of human primary brown adipocytes upon stimulation with norepinephrine (10 μM) or different concentrations of ATR-127 (N = 8–14 replicates derived from 2 different patients). (E) Quantification of oxygen consumption rates calculated upon compound addition as increase over OG % response. (F) Gene expression levels of UCP1 in human BAT cells upon PBS and ATR-127 treatment (N = 3) (G) *in vivo* basal oxygen consumption upon acute injection of ATR-127 (5 mg/kg) in C57Bl/N6 mice. (H) *in vivo* average light phase energy expenditure of C57Bl/N6 mice upon acute injection with ATR-127 (5 mg/kg). (I) *In vivo* glucose uptake in brown adipose tissue of C57Bl/N6 mice upon acute injection with insulin (1 mg/kg), ATR-127 (1 mg/kg), or CL-316,243 (1 mg/kg). One data point was removed in Figure 3I based on outlier test. In the event of normal distribution, data were analyzed by means of the Student's t test followed by Mann Whitney test or one-way ANOVA followed by the Dunnett's or Sidak's multiple comparison tests. When the data deviates from normal distribution, it underwent analysis using the Kruskal–Wallis test, followed by Dunn's multiple comparisons.∗p < 0.05, ∗∗p < 0.01, ∗∗∗∗p < 0.0001. (h) BAT = (human) brown adipose tissue, OCR = oxygen consumption rate, OG = Oligomycin, CPD = Compound (ATR-127 or NE), NE = norepinephrine, CL = CL-316,243. (For interpretation of the references to color in this figure legend, the reader is referred to the Web version of this article.)

We next investigated the effects of ATR-127 on glucose uptake and thermogenesis in different adipose tissue depots. We previously showed that isolated mature brite adipocytes from 3-week-old NMRI mice produced quantifiable amounts of heat after adrenergic receptor stimulation, despite relatively low amounts of Ucp1 [45]. For this reason, we performed microcalorimetry on mature inguinal brite adipocytes treated with a positive control, CL-316,243 (1 μM), or ATR-127 (1 μM). Importantly, both CL316243 and ATR-127 resulted in a significant increase in cumulative heat production in isolated brite adipocytes (Figure 3A–B).

Next, we investigated the effects of ATR-127 on BAT metabolism. We observed increased glucose uptake in primary mouse BAT cells upon acute treatment with ATR-127, that was indistinguishable from that to NE and the selective β_3_ agonist CL-316243 (p ≤ 0.001, Figure 3C). In addition, we found that ATR-127 increased oxygen consumption rates in human BAT cells (Figure 3D–E), indicating activation of thermogenesis. This was further supported by significant ATR-127 (10 μM) -mediated induction of uncoupling protein 1 (UCP1) expression (p = 0.05, Figure 3F), a key mediator of BAT thermogenesis. Acute injection of ATR-127 (5 mg/kg) in C57Bl/N6 mice also significantly increased basal oxygen consumption rate and light phase energy expenditure (p ≤ 0.0001 and p = 0.027, respectively, Figure 3G–H). In addition, we observed significant increase in overall 24 h oxygen consumption (p ≤ 0.0001, Suppl Fig 6). The observed effects on energy expenditure, primarily attributed to BAT thermogenesis, were accompanied by elevated BAT tissue glucose uptake (p ≤ 0.0001, Figure 3I). In contrast, incubation of white adipocytes with ATR-127 *in vitro* did not affect oxygen consumption rates (Supplementary Figure 2A-B), although acute injection resulted in moderately increased glucose uptake in white adipose tissue (WAT) in C57Bl/N6 mice (Supplementary Figure 2C). Combined, these results strongly suggest that ATR-127 activates thermogenesis and increases glucose uptake, particularly in BAT.

### 2.4 Chronic ATR-127 treatment improves glucose tolerance and body composition in DIO mice

**Figure 4:**
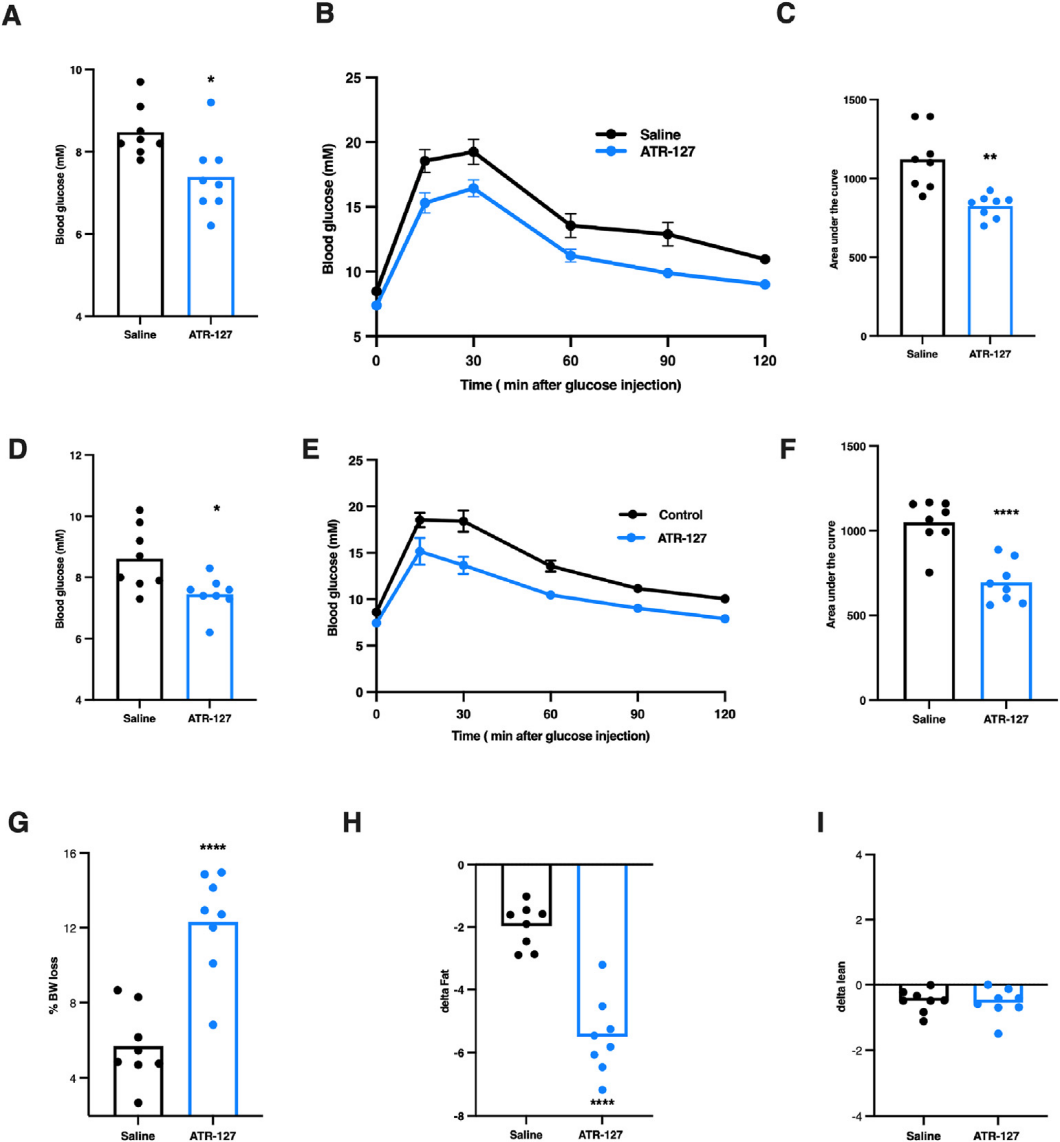
**Prolonged ATR-127 treatment improves glucose homeostasis and reduces fat mass in diet-induced obese mice.** (A) Fasting blood glucose upon 4-days of ATR-127 treatment (5 mg/kg). (B) Intraperitoneal glucose tolerance test following 4-days of ATR-127 treatment (5 mg/kg). (C) area under the curve of intraperitoneal glucose tolerance test (5 mg/kg). (D) Fasting blood glucose upon 11-days of ATR-127 treatment (5 mg/kg). (E) Intraperitoneal glucose tolerance test following 11-days of ATR-127 treatment (5 mg/kg). (F) area under the curve of intraperitoneal glucose tolerance test (5 mg/kg). (G) Percentage body weight loss upon 21 days of ATR-127 treatment (5 mg/kg). (H) Delta fat mass change. (I) Delta lean mass change. N = 8 for all experiments. In the event of normal distribution, data were analyzed by means of a Student's paired *t* test. ∗p < 0.05, ∗∗p < 0.01, ∗∗∗p < 0.001, ∗∗∗∗p < 0.0001. BW = body weight.

Encouraged by these positive acute effects, we next examined the effects of more prolonged ATR-127 treatment on glucose homeostasis and body composition in DIO mice. Daily intraperitoneal (i.p) injection with ATR-127 (5 mg/kg) for 3-weeks in DIO mice (Suppl Figure 3) significantly decreased fasting blood glucose levels (p ≤ 0.01) and improved glucose tolerance as early as 4 days treatment (AUC: p ≤ 0.001, Figure 4A–C). These effects on fasting blood glucose and glucose tolerance were maintained following 11 days of treatment (Figure 4D–F). In line with fasting blood glucose levels, fasting insulin levels were significantly reduced by 66% after 21 days of treatment (p ≤ 0.001) (Suppl Figure 4A). Plasma free fatty acids were elevated by 32% upon prolonged ATR-127 treatment, suggesting stimulation of lipolysis (p = 0.08) (Suppl Figure 4B). Total cholesterol levels were not affected by ATR-127 treatment (Suppl Figure 4C). Body weight and fat mass gradually decreased over time during treatment, despite no differences in food intake (Suppl Figure 5A-C). After 21 days of treatment, both body weight and fat mass were significantly reduced (p < 0.0001, Figure 4G–H), whereas no significant differences were observed in lean mass (Figure 4I and Suppl Figure 5D).

### 2.5 Chronic ATR-127 treatment increases BAT thermogenesis

**Figure 5:**
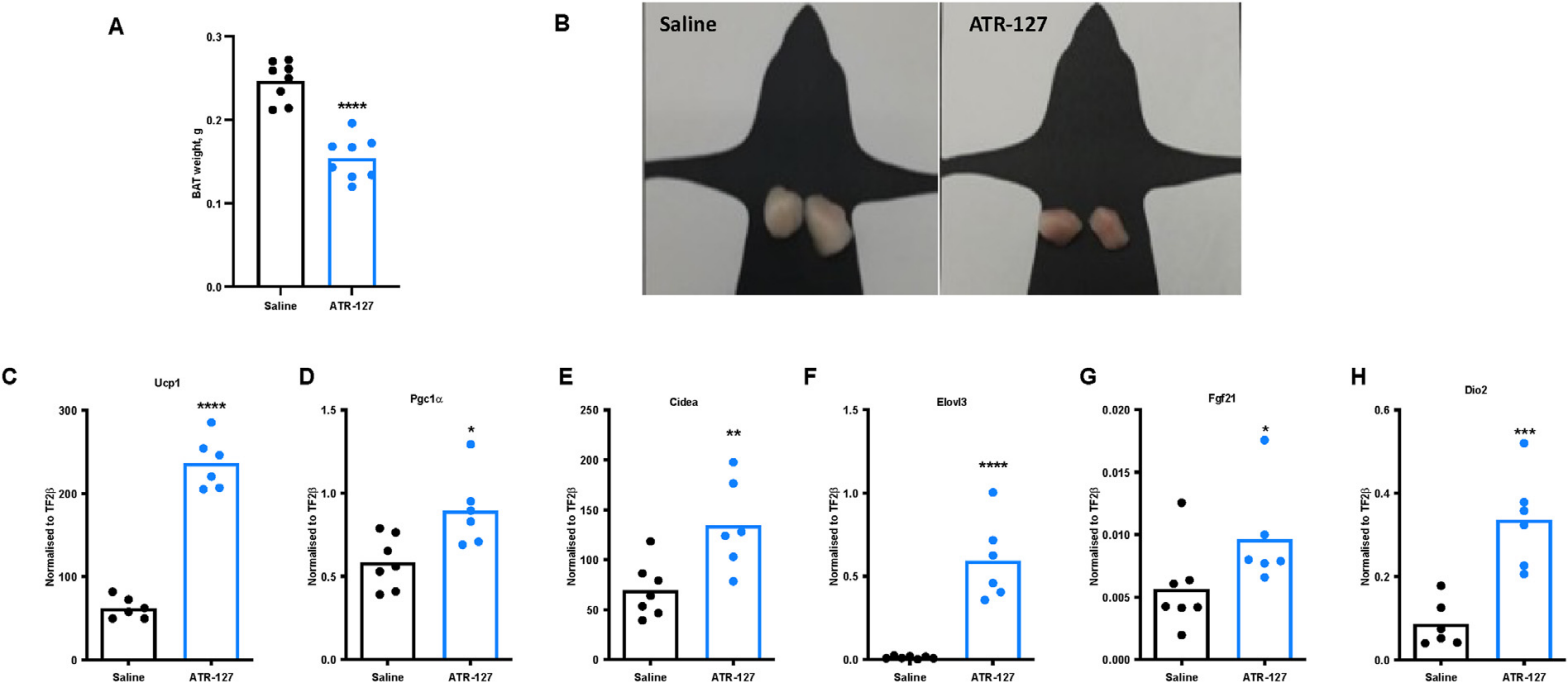
**Prolonged ATR-127 treatment induces BAT browning in diet-induced obese mice.** (A) Brown adipose tissue weight following 21-days of ATR-127 treatment. (B) Brown adipose tissue depots (C-H) Gene expression of Ucp1, Pgc1a, Cidea, Elovl3, Fgf21 and Dio2 normalized to the expression of TF2-beta in brown adipose tissue (N = 6–7). One data point was removed in Figure 5C,H based on outlier test. In the event of normal distribution, data were analyzed by means of a Student's paired *t* test. When the data deviates from normal distribution, it underwent analysis using Mann-whitney test. ∗p < 0.05, ∗∗p < 0.01, ∗∗∗∗P < 0.0001. BAT = brown adipose tissue. (For interpretation of the references to color in this figure legend, the reader is referred to the Web version of this article.)

To further explore the underlying mechanisms of ATR-127-mediated improvements in glucose homeostasis and body weight/composition, we focused on the effects on BAT as it accounts for most β-AR agonist-induced increases in whole-body energy expenditure [46]. Upon sacrifice, mice treated with ATR-127 (5 mg/kg) for 21 days showed a marked reduction in BAT tissue weight and increased browning (Figure 5A–B). This was paralleled by a significant upregulation of the key thermogenic genes Ucp1, Pgc1a, Cidea, Elovl3, Fgf21 and Dio2 (Figure 5C–H). Combined, these results indicate that ATR-127 may increase thermogenic capacity.

### 2.6 ATR-127 reduces hepatic steatosis

**Figure 6:**
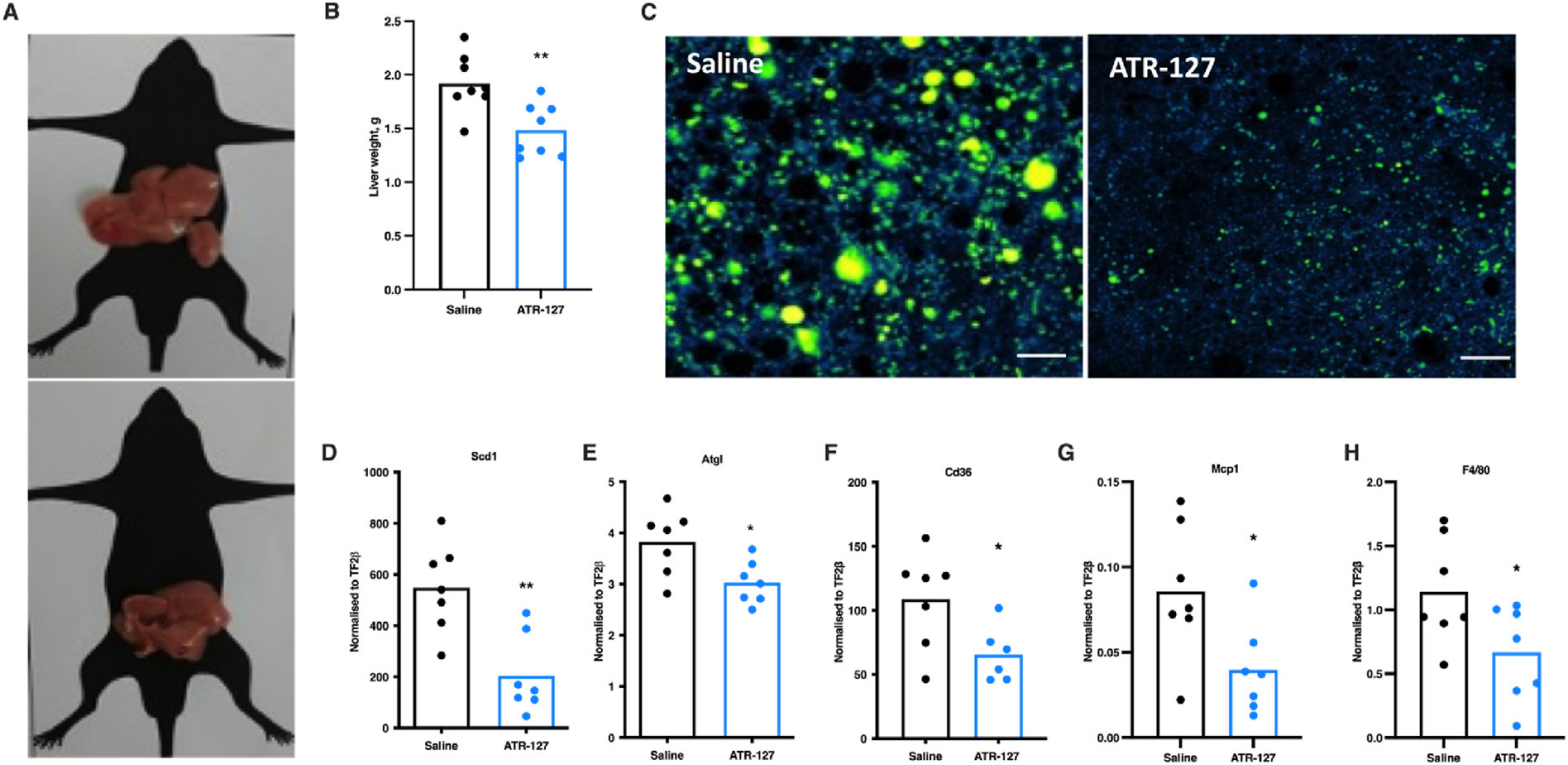
**ATR-127 treatment for 21 days reduces hepatic steatosis in diet-induced obese mice** (A) Liver morphology. (B) Liver weight. (C) Hepatic lipid staining with BODIPY (green), (scale bar = 50 μm). (D–H) Hepatic gene expression of Scd1, Atgl, Cd36, Mcp1, and F4/80 normalized to TF2β expression (N = 7). One data point was removed in Figure 5G based on outlier test. In the event of normal distribution, data were analyzed by means of a Student's paired *t* test. A significant difference was considered at ∗p < 0.05, ∗∗p < 0.01. (For interpretation of the references to color in this figure legend, the reader is referred to the Web version of this article.)

Liver weights were reduced upon prolonged ATR-127 (5 mg/kg) treatment (p ≤ 0.001, Figure 6A–B) and this was primarily associated with a reduced BODIPY staining, indicating qualitative differences in lipid content between the control and treated groups (Figure 6C). Analysis of gene expression in liver of key enzymes involved in lipid metabolism (Atgl), synthesis of triacylglycerols (Scd1) and fatty acid uptake (Cd36) showed reduction in treated animals compared to controls (Figure 6D–F). We also observed reduced expression of inflammation-related genes (Mcp1 and F4/80) in the liver of treated mice (Figure 6G–H). Combined, these data demonstrate the efficacy of ATR-127 in reducing hepatic steatosis in DIO mice.

### 2.7 ATR-127 demonstrates cardiac safety in DIO mice and in human heart strips *ex vivo*

**Figure 7:**
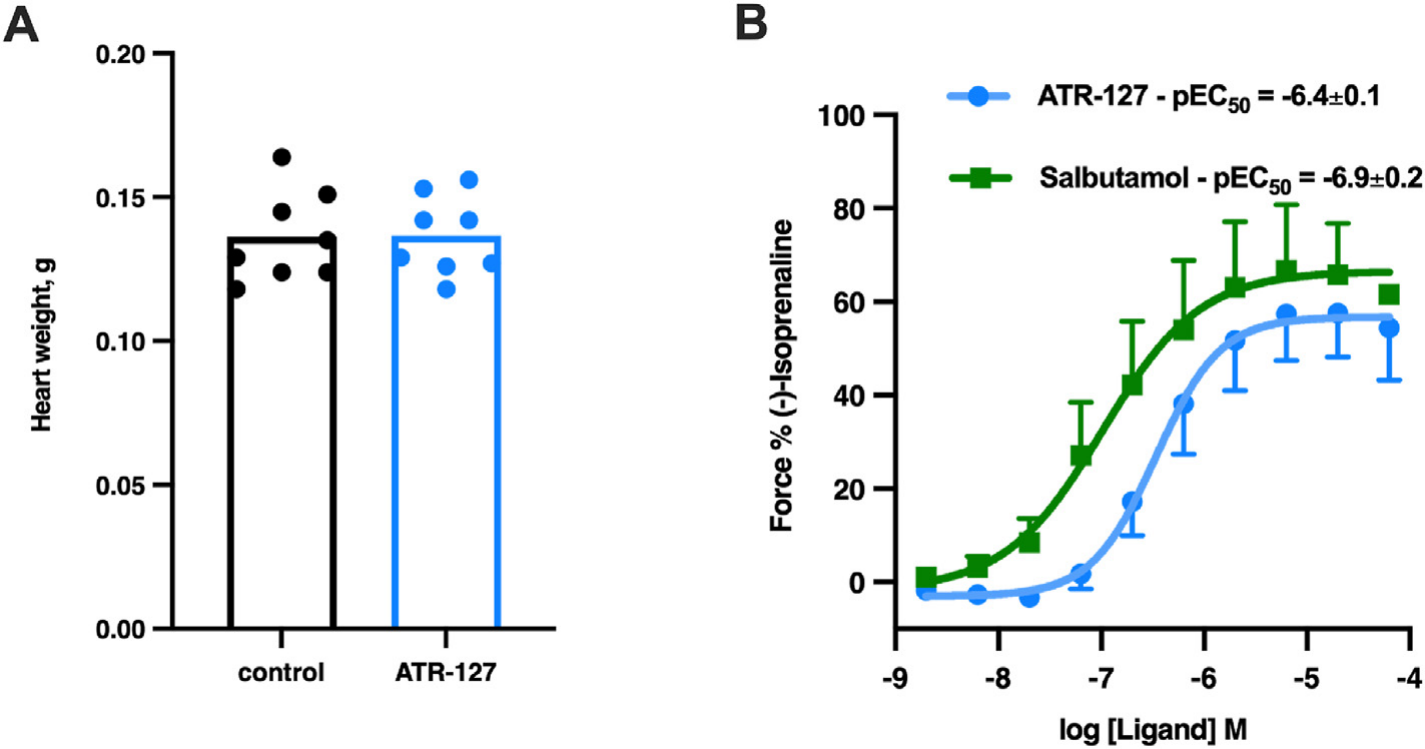
**Effects of prolonged treatment with ATR-127 on mouse heart weight and ex vivo in human heart strips.** Diet-induced obesity was developed in C57Bl/6N mice maintained at 30 °C and on HFD for 4 months; DIO mice were treated daily with 5 mg/kg ATR-127 for 3 weeks (n = 8). (A) heart weight. (B) Mean cumulative concentration-response curves to Salbutamol and ATR-127 in 5 electrically driven human right atrial appendage trabeculae from 4 patients. Inotropic responses are expressed as a percentage of the maximum response to (−)-isoprenaline. Each data point represents mean ± SEM.

Lastly, to explore if ATR-127 has any potential to adversely affect hearts, we performed a basic assessment of heart weight in DIO mice and did not observe any difference between the treated and untreated groups, implying that ATR-127 does not induce cardiac hypertrophy (Figure 7A). Furthermore, in an *ex vivo* study utilizing 5 human right atrial trabeculae from 4 patients, we evaluated cardiac contractility and force generation. ATR-127 generated 41.2% less force than isoprenaline, similar to the effects of salbutamol (Figure. 7B). The pEC50 value for ATR-127 is −6.35 ± 0.11, making it approximately 100 times less potent than isoprenaline, which has a pEC50 of −8.48. Additionally, the maximal contractile effect of ATR-127 (58.8 ± 8.8% of the maximal force of isoprenaline) is lower than that of salbutamol (70.5 ± 11.3% of the maximal force of isoprenaline), with a mean EC50 value of log −6.9 ± 0.2 (Figure. 7B). ATR-127 clearly exhibits significantly reduced positive inotropic effects compared to isoprenaline. This data is in line with reduced cAMP induction in rat cardiomyocytes compared to isoprenaline (Figure. 1E).

## 3 Discussion

Activation of skeletal muscle β_2_-ARs or BAT β_3_-ARs has previously shown beneficial effects on metabolic health in rodents [25,30,37], suggesting that dual activation of these receptors could be used additively or synergistically to treat obesity and its comorbidities in humans. Although highly promising, activation of β_2_-or β_3_-ARs by currently available agonists used therapeutically, is associated with cardiovascular side effects rendering them unsuitable for this clinical application. In the current study, we have examined the therapeutic potential of our newly developed β_2_-β_3_-AR dual agonist ATR-127 that induces low intramyocellular cAMP, yet retains many of the desired metabolic effects. Incubation of L6 cells with ATR-127 markedly increased skeletal muscle glucose uptake *in vitro*, despite producing lower intramyocellular cAMP concentrations than isoprenaline, and a similar effect on glucose uptake was observed *in vivo*. In addition, ATR-127 robustly enhanced BAT thermogenesis and glucose uptake both in primary cell culture and *in vivo*. Chronic ATR-127 treatment (5 mg/kg/day for 3 weeks) also significantly improved whole-body glucose homeostasis and reduced body weight and fat mass in DIO mice. These effects were associated with increased BAT thermogenic capacity and reduction of hepatic steatosis. Moreover, ATR-127 exhibited minimal cardiovascular side effects in both *in vivo* and *ex vivo* experiments. Combined, these results strongly suggest that a dual partial agonist such as ATR-127 could be used as a novel therapeutic approach to combat obesity and associated metabolic disorders.

β-ARs are members of the adrenoceptor GPCR family that are widely recognized as important therapeutic targets for the treatment of a variety of diseases [47]. However, prolonged systemic stimulation of β_2_-or β_3_-ARs in humans has proven to be challenging, not only because of the pharmacological profile, but also due to cardiovascular side effects and the occurrence of tachyphylaxis. These adverse effects of high efficacy, long-acting β-AR agonists result in large increases in intramyocellular cAMP levels and recruitment of β-arrestins [48] upon activation. Interestingly, the signaling pathways underlying these unfavorable events appear to be ligand selective and are associated with distinct conformations of the β-AR upon ligand binding [47,49]. This suggested that a β_2_-β_3_-AR dual agonist could be developed with beneficial health effects whilst simultaneously limiting adverse events, leading to the creation of our first-in-class small-molecule adrenergic agonist ATR-127. In line with our hypothesis, ATR-127 demonstrated engagement of G_s_, G_15_, and GRK/Gβγ signaling, thereby confirming selective activation of particular intracellular signaling pathways. This signaling pattern was associated with lower desensitization and reduced levels of intramyocellular cAMP. However, future studies should be initiated performing in-depth experiments focusing on the desensitizing effects of ATR-127 *in vivo*. To our knowledge, this is the first β_2_-β_3_-AR agonist with potential to improve metabolic health with minimal cardiovascular side effects and with less internalization, suggestive of reduced tachyphylaxis. Although highly promising, it is important to note that ATR-127 still weakly activates β_1_-ARs that could induce off-target cardiovascular effects [50,51]. However, chronic ATR-127 treatment (5 mg/kg/day for 21 days) did not affect heart weight in DIO mice, indicating that cardiac hypertrophy did not occur. Furthermore, when we explored this phenomenon in a human context using an *ex vivo* model with human cardiac tissue, we observed a notably lower contractile force than that of isoprenaline [52], highlighting its reduced impact on cardiac contractility. Moreover, the maximal contractile effect of ATR-127 was less than salbutamol, indicating that ATR-127 is also a partial β-AR agonist. Salbutamol is a partial agonist of β_2_-AR in the human heart [53], displaying approximately a 20-fold higher affinity for β_2_-AR compared to β_1_-AR [54]. Evidence suggests that direct administration into the right coronary artery of patients with stable angina results in elevated heart rate [55], while inhalation via nebulizer in individuals with asthma or COPD leads to increased heart rate and atrioventricular (AV) node conduction [56]. However, a meta-analysis encompassing β2 adrenergic receptor agonist treatment, including salbutamol and the non-selective β-AR agonist isoprenaline, in patients with asthma or COPD indicated a rise in heart rate, with a “non-significant” increase in adverse cardiovascular events such as ventricular tachycardia and sudden death [57]. Given the potent vasodilatory properties associated with β2 agonists, they may hold promise in managing pulmonary hypertension. In addition, this finding aligns with the data we gathered from the mouse experiments. The cumulative findings from both mouse and human studies provide compelling evidence that the positive inotropic effects of ATR-127 are significantly lower when compared to isoprenaline, further reinforcing its potential relative safety as a therapeutic agent. However, caution may be warranted in patients with obesity and cardiovascular disease, especially those at risk of arrhythmias, pending further safety evaluations, particularly for drugs like ATR-127.

The development strategy for new medications targeting diabetes and obesity involves leveraging β_2_- and β_3_-adrenergic pathways with minimal cardiovascular risk. ATR-127, identified as a partial agonist compared to isoprenaline, demonstrates a lower maximal inotropic effect. Additionally, there may be benefits if ATR-127 can be administered at a low dose sufficient to achieve therapeutic effects on skeletal muscle and adipose tissue targets while minimizing potential adverse effects on the heart.

In human subjects, β_1_ - AR activation is associated with adverse outcomes, while the impact of β_2_-AR stimulation is more intricate and has been linked to positive effects [58]. However, further comprehensive studies, including clinical trials, are warranted to validate these initial findings and assess the translational potential of ATR-127 as a viable therapeutic option.

Previous studies show that selective β_2_- or β_3_-AR agonists have great promise for the treatment of obesity owing to their intrinsic capacity to enhance energy expenditure in humans upon both acute and chronic treatments [16,21,[59], [60], [61]]. Based on these characteristics, it could be hypothesized that a selective dual agonist could additively, or even synergistically, enhance energy expenditure in patients with obesity, thereby inducing weight loss and improving metabolic health. In line with this notion, we have demonstrated that ATR-127 not only robustly increased energy expenditure upon acute injection in healthy mice, but also significantly reduced body weight and fat mass in DIO mice upon chronic treatment. Importantly, these effects were not associated with skeletal muscle breakdown, which is considered a key side-effect of GLP1 agonists [62,63], as we could show lean mass retention. Although some clinical studies have investigated the effects of treatment with a selective β_2_- or β_3_-AR agonist on weight loss and fat mass, the results have been somewhat contradictory [18,21,59], potentially due to some studies being conducted in healthy individuals or performed over short periods of time. As such, future studies with ATR-127 should focus on the effects on body weight and fat mass in patients with obesity over a prolonged period of time.

The thermogenic effects of selective β_2_- or β_3_-AR agonists are associated with upregulation of energy-consuming processes in skeletal muscle and BAT [16,64,65]. Although we have not explored the effects of ATR-127 on skeletal muscle thermogenesis in detail, it appears likely that skeletal muscle is involved in ATR-127-mediated increases in energy expenditure. For example by stimulation of Na^+^/K^+^ ATPase-pump activity [66], UCP3-mediated uncoupling of mitochondrial respiration [67] or the futile Ca^2+^ cycle upon β_2_-AR activation [21] Nevertheless, BAT also has a great capacity to increase energy expenditure by UCP1-mediated uncoupling of oxidative phosphorylation following β_3_-AR stimulation [32]. In line with this, ATR-127 significantly increased BAT cells oxygen consumption *in vitro*, increased whole-body oxygen consumption upon acute injection in C57Bl/N6 mice, and increased markers of BAT tissue browning in DIO mice upon prolonged treatment. These findings are highly intriguing, since humans have been well-characterized to possess thermogenically functional BAT depots in adulthood, both in healthy and in obese individuals [[38], [39], [40]]. Although pharmacological activation of these BAT depots with selective β_3_-AR agonists has been achieved at high doses [59,65], these effects could also be explained by off-target effects at β_2_-AR associated with human BAT activation [41,42]. We have shown here that ATR-127 treatment enhanced thermogenesis and the expression of the key thermogenic gene UCP1 in human primary brown adipocytes, an effect potentially related to the effects of ATR-127 at both β_2_- and β_3_-AR suggesting that ATR-127 may be more effective in stimulating human BAT than previously investigated compounds. Although these data are highly promising, future studies should focus on the *in vivo* effects of ATR-127 on BAT thermogenesis.

Another important incentive for the development of a novel β_2_-β_3_-AR agonist relates to its potential to counter T2DM, commonly associated with obesity [68]. More specifically, preclinical studies have demonstrated marked improvements in glucose homeostasis upon prolonged treatment with selective β_2_- or β_3_-AR agonists by stimulation of glucose uptake in skeletal muscle or BAT [[24], [25], [26],^30^,^35^,^36^]. Here we found that treatment of DIO mice with ATR-127 for 21 days robustly decreased fasting plasma glucose and insulin concentrations and improved glucose tolerance. Combined with our observed *in vitro* effects of ATR-127, it seems reasonable to conclude that these effects are largely due to increased glucose uptake in skeletal muscle and BAT tissue. From a clinical perspective, the effects of ATR-127 on skeletal muscle glucose uptake are highly intriguing, as blunted glucose uptake in muscle is a primary hallmark associated with the development of T2DM [69]. [26] Furthermore, β_2_-AR-mediated skeletal muscle glucose uptake occurs independently [24,26], thereby bypassing defects in intracellular insulin signaling commonly seen in T2DM. The clinical significance of this phenomenon has been corroborated in various clinical studies, with our own research [21], as well as those of other investigators [61,70] showing substantial enhancements in insulin-stimulated glucose uptake within peripheral tissues, particularly skeletal muscle, following extended treatment with a β2-AR agonist in healthy young men. An increase in skeletal muscle glucose uptake, which operates independently of insulin signaling, could therefore result in an improvement of insulin sensitivity. Combined, these findings suggest that ATR-127 will induce similar – or even more pronounced effects in individuals with impaired glucose homeostasis. As such, future studies should also focus on investigating potential anti-diabetic effects of ATR-127 in humans.

The liver also appears to play a pivotal role in ATR-127-mediated improvements in glucose homeostasis. Our observed decreases in fasting plasma glucose concentrations are likely attributable to an improvement in hepatic insulin sensitivity and, thereby, a reduction in hepatic glucose release [71]. These improvements in hepatic insulin sensitivity could be caused by a reduction in hepatic steatosis a well-known factor to be associated with hepatic insulin resistance [72,73]. Indeed, after ATR-127 treatment, we demonstrated a decrease in liver weights. While we cannot discount the contribution of a reduction in liver glycogen content to this effect, as we have shown previously in clenbuterol treated DIO mice [25], a qualitative decrease in lipid content assessed through BODIPY staining was indicative of a reduction in hepatic steatosis. Additionally, while total inflammation was not specifically measured, the decrease in liver weight was accompanied by a concomitant decrease in inflammatory and adipogenic gene expression further suggesting an improvement in steatotic parameters. We have also demonstrated that prolonged treatment with the β_2_-AR agonist clenbuterol drastically reduced hepatic lipid accumulation in DIO mice [25]. These findings suggest that adrenergic agonists, such as ATR-127 improves hepatic glucose handling, but also potentially combats NAFLD in patients with obesity.

Current approaches by the pharmaceutical industry have primarily focused on developing anti-obesity medication capable of reducing food consumption or nutrient uptake, thereby shifting the body's energy balance towards a caloric deficit and thus inducing weight loss. Thus, GLP-1 agonists and SGLT-2 inhibitors, that induce weight loss in overweight and obese individuals [74] or T2DM [75], pave the way for further clinical applications. Nevertheless, it is important to note that one side effect of these drugs is loss of skeletal muscle [62,63], that could potentially lead to the development of other metabolic disorders after prolonged use. Thus, ATR-127 since it has an intrinsic ability to affect other aspects of energy balance (i.e. energy expenditure) whilst simultaneously preserving lean mass due to its β_2_-AR agonism, appears to be a highly attractive, first-in-class therapeutic compound. These characteristics highlight the potential of ATR-127 as a therapeutic compound to target obesity and associated metabolic perturbations, that should be further investigated in a clinical setting.

In conclusion, we demonstrate that our newly developed adrenergic agonist ATR-127 can improve glucose homeostasis via both β_2_-and β_3_-ARs, with minimal receptor internalization, and low intramyocellular cAMP and minimal cardiovascular side effects (including contractility). Treatment of DIO mice with ATR-127 was highly effective at reducing body weight and fat mass, whilst preserving lean mass. Besides these effects on body weight, ATR-127 treatment significantly improved whole-body glucose homeostasis, presumably by increased glucose uptake into skeletal muscle and BAT tissue, as well as reducing hepatic steatosis (Graphical abstract). These results suggest that ATR-127 is a promising novel therapy to combat obesity and its comorbidities in humans.

## 4 Methods

### 4.1 Reagents

D-PBS, Dulbecco's Modified Eagle Medium, Trypsin, PBS, penicillin and streptomycin, fetal bovine serum (FBS) and Poly-D-Lysine (PDL) were purchased from Gibco (Thermo Fisher Scientific, Waltham, MA, USA). Polyethylenimine (PEI) was purchased from Alfa Aesar (Thermo Fisher Scientific, Waltham, MA, USA). Coelenterazine 400a was purchased from Nanolight Technologies (Pinetop, AZ, USA).

### 4.2 ATR-127 chemical synthesis

The synthesis of ATR-127 has previously been described [76,77] and an improved schema is depicted in Figure 1. In the bromination reaction of 3-benzyloxyacetophenone (**1**) a mixture of difficult to separate mono- and di-brominated products were typically obtained, however the treatment of the crude mixture with diethyl phosphite allowed for the desired 3-benzyloxyphenacyl bromide to be obtained conveniently. In the next step, reduction using the Corey–Bakshi–Shibata (CBS) conditions was employed to obtain (R)-bromoalcohol **2** with high enantioselectivity (99:1 er). Its treatment with K_2_CO_3_ in methanol gave the epoxide **3** that was used in the reaction with (R)-N-((R)-1-phenylethyl)pentan-2-amine to afford compound **4** in high yield. Two equivalents of epoxide reagent **3** and 2 eq of *i-*PrOH as the additive were required to complete the conversion of the secondary amine, thus avoiding laborious separation of the desired product from the unreacted starting amine. Simultaneous reductive deprotection of both amine and phenol groups followed by hemisulfate salt formation gave the final product ATR-127.

### 4.3 Construct generation

The β_2_-AR construct was generated by amplifying the full length sequence of SNAP-tag (New England Biolabs, Ipswich, MA) and fusing it in frame with the CD8 membrane signal sequence within pcDNA3.1 (ThermoFisher, Waltham, MA) to yield sig.SNAP. The full length coding sequences of human β_2_AR (with the initial methionine removed) was added to the 3’ end the sig.SNAP in pcDNA3.1 to give the constructs designated as SNAP-β_2_AR. The sequence was confirmed by Sanger sequencing.

Plasmaid DNA constructs of p63RhoGEF-*R*lucII, Rap1Gap-*R*lucII, PDZRhoGEF-*R*lucII [78] GRK2-D110A-*R*lucII [79], β-arrestin-2-*R*lucII [80], and rGFP-CAAX [81] have been described previously. β_2_AR, Gα_i1_, Gα_i2_, Gα_i3_, Gα_oA_, Gα_oB_, Gα_z_, Gα_q_, Gα_11_, Gα_14_, Gα_15_, Gα_12_ and Gα_13_ were purchased from cDNA.org (Bloomsburg University, Bloomsburg, PA). *R*luc8-mGs was generated by subcloning from NES-Venus-mGs into *R*luc8-C1 vector by using EcoRI and XhoI. All plasmid constructs were verified by Sanger sequencing.

### 4.4 Cell cultures

#### 4.4.1 Chinese hamster ovary cell cultures

Immortalized Chinese hamster ovary (CHO) cells stably expressing the mouse β_3-_AR (CHO mβ_3-_AR) were grown in DMEM/F12 medium (Gibco, Cat#105650s18) containing 10% (v/v) fetal bovine serum (FBS) and 1% penicillin and streptomycin (Pen/Strep; Gibco, Cat#15070063) at 37 °C in 5% CO_2_. The cells were grown until reaching ≈90% confluence and were split twice a week. After every 5 passages, cells were treated in growth medium containing G418 (0.8 mg/mL; Invivogen, Cat#ant-gn-5). For cAMP assays, cells were plated at 1 × 10^4^ cells/well overnight in 96 well plates and were serum starved overnight.

#### 4.4.2 Skeletal muscle cell cultures

L6 muscle cells stably expressing GLUT4-myc (Professor Amira Klip, Hospital for Sick Children, Toronto, ON, Canada) were grown in DMEM supplemented with 4 mmol/L L-glutamine, 10% FBS, 100 μg/mL streptomycin, 100 units/mL penicillin and 10 mmol/L HEPES in a 37 °C incubator with 8% CO_2_. The cells were grown to ∼90% confluence with differentiation induced by reducing FBS to 2% for 7 days.

#### 4.4.3 Neonatal rat ventricular myocyte (NRVM) culture

Protocols were approved by the Monash Institute of Pharmaceutical Sciences Animal Ethics committee, abiding by the Australian code for the care and use of animals for scientific purposes. 1–2 days old Sprague Dawley rats of either sex was used, and rats killed by decapitation. The hearts were removed, and ventricles dissected and incubated in Hanks Balanced Salt Solution (HBSS; ThermoFischer Scientific, Cat#14065-056) containing 0.1% trypsin at 4 °C overnight with constant stirring. The trypsin was deactivated with addition of DMEM high glucose containing 10% FBS. Cells were further dissociated by the addition of type II collagenase (2.5 mg/ventricle; Worthington Biochemical Corp) at 37 °C in a shaking incubator (10 min, 100 rpm). The dissociated cells were collected by centrifugation at 400 g for 5 min and resuspended in DMEM containing 10% FBS. Cells were pre-plated on 150 mm culture dishes for 1 h at 37 °C to remove any fibroblasts. The nonadherent cells (myocytes) were then transferred to another dish for another 1 h, before the non-adherent cells were counted using a hemocytometer. Cells were plated into 96-well plates at 3 × 10^4^ cells/well and incubated in DMEM high glucose containing 10% FBS, 100 mM 5-bromo-2-deoxyuridine and 1% Pen/Strep overnight to stop any cardiac fibroblast growth. Cells were then maintained in DMEM for up to 5 days. Before being used for cAMP assays, cells were serum starved overnight.

#### 4.4.4 Human embryonic kidney (HEK 293A) cell culture

HEK 293A cells were purchased from Thermo Fisher Scientific (RRID: CVCL_6910) and maintained in DMEM supplemented with 10% FBS, 100 U/mL penicillin and 100 μg/mL streptomycin (For β_2-_AR internalization assay cells were cultured in DMEM high-glucose). Cells were grown at 37 °C in 5% CO_2_ and 90% humidity and were regularly checked for mycoplasma contamination.

#### 4.4.5 Mouse primary adipocyte cultures

Three to four weeks old NMRI mice (Charles River, Code 249800-801) were euthanized by CO_2_, and pooled interscapular, cervical and axillary brown adipose tissue depots (iBAT, cBAT and sBAT) and inguinal WAT tissue were dissected. Tissues were minced with scissors in DMEM and transferred to a collagenase buffer (0.2% collagenase (type II, Gibco) containing 0.1 M HEPES, 123 mM NaCl, 5 mM KCI, 1 mM CaCl_2_, 4.5 mM glucose and 1.5% BSA) and digested for 30 min at 37 °C. The digested tissue was filtered through a 250 μm filter, and the filtrate was put on ice for 20 min to allow the mature adipocytes to float. The infranatant was filtered through at 50 μm filter and centrifuged (10 min, 1000×*g*). The pellet, containing the stromal vascular fraction, was resuspended in high glucose DMEM preheated to 37 °C and centrifuged again at 1000×*g* for 10 min. The pellet was resuspended in culture medium (DMEM, 4.5 D-glucose/L, 10% newborn calf serum, 4 nM insulin, 10 mM HEPES, 4 mM glutamine, 25 μg/mL sodium ascorbate, 50 U/mL penicillin and 50 μg/mL streptomycin) and the cells were plated (day 0). Plates were kept at 37 °C with 8% CO_2_, culture medium was changed on day 1, 3 and 5 of differentiation and the cells were used for CalScreener or glucose uptake assays on day 7.

#### 4.4.6 Human primary adipocyte cultures

Subcutaneous white adipose tissue and Brown adipose tissues were obtained from the patients of thyroid surgery with a normal thyroid function and cultured as described earlier (Broeder et al.). Procedure was reviewed and approved by the ethics committee of Maastricht University Medical Center (METC 10-3-012, NL31367.068.10).

For geneexpression analysis, preadipocytes were isolated and cultured from human supraclavicular adipose tissue as previously described [82]. Cells were grown in DMEM/F12 with 10% FBS, 1% Penicillin-Streptomycin, and 1 nM FGF-1 at 37 °C with 5% CO2. The culture medium was refreshed every other day. Adipocyte differentiation was initiated two days after preadipocytes reached confluence, using DMEM/F12 containing 1% Penicillin-Streptomycin, 0.1 μM dexamethasone, 100 nM insulin, 200 nM rosiglitazone, 540 μM IBMX, 2 nM T3, and 10 μg/mL transferrin. IBMX was removed after three days, and the cultures continued differentiation for an additional nine days with medium changes every third day. Rosiglitazone was removed on differentiation day 6. On the experimental day, cells were serum-starved for 2 h before stimulation. Cells were stimulated with 10 μM norepinephrine and 10 μM ATR-127 for 4 h, after which RNA was harvested using Trizol (Ethical approval number:H-A-2009-020).

#### 4.4.7 Human right atrial trabeculae tissue harvesting and processing *ex vivo*

Right atrial appendage used in this study was collected from patients undergoing cardiac surgery at The Prince Charles Hospital (TPCH). Patients provided written informed consent before coronary artery by-pass surgery, aortic root and/or valve replacement. The study was approved by the Metro North Hospital and Health Services Human Ethics Committee according to the Declaration of Helsinki, approval references HREC/12/QPCH/275.

Patient characteristics are outlined in Table 1. Following excision, right atrial cardiac tissue was immediately immersed in ice-cold pre-oxygenated modified Krebs solution containing (mM); Na^+^ 125, K^+^ 5, Ca^2+^ 2.25, Mg^2+^ 0.5, Cl^−^ 98.5, SO_4_^2−^ 0.5, HCO^3−^ 32, HPO_4_^2-^1, ethylenediaminetetraacetic acid 0.04, and equilibrated with 95% O2/5% CO2 and transported to the laboratory. Intact atrial trabeculae were dissected and placed in a 50 mL organ bath containing modified Krebs solution and attached to Swema SG4-45 strain gauge force transducers connected to a PowerLab Data acquisition system using LabChart Version 8.1.13 recording software. Atrial trabeculae were stimulated with square-wave pulses of 5 ms duration just above threshold voltage to contract at 1 Hz at 37 °C. A length-tension curve was constructed to determine the optimal length at which maximal contraction occurred (*L*max) and then adjusted to 50% of the force observed at *L*max. The incubation medium was exchanged with 50 mL of modified Krebs supplemented with amino acids: Na^+^ 15 mM, fumarate 5 mM, pyruvate 5 mM, L-glutamate 5 mM, glucose 10 mM. Trabeculae contractile force reached a steady state prior to the commencement of all experiments. To determine the potency and efficacy of ATR-127 and Salbutamol, trabeculae were setup and reached a steady state before the commencement of a concentration effect curve by sequential administration of ½ log increments commencing at 2 nM to 60 μM, followed by 200 μM of (−)-isoprenaline. The maximal force obtained at each concentration was used to construct a concentration-effect curve from which the logEC_50_ (logEC_50_) value was obtained.

**Table 1:** Patient Characteristics.

Drugs	ATR-127	Salbutamol
No. of Patients	4	4
Age	59.8 ± 9.6	64.0 ± 4.2
**Gender**		
Male	4 (100%)	4 (100%)
**Procedure**		
CABG	3 (75%)	4 (100%)
CABG + Aortic Root Replacement	1 (25%)	–

### 4.5 cAMP assay experiments

CHO cells were incubated with varying concentrations of ATR-127 or isoprenaline (Sigma–Aldrich, Cat#I2760-100 MG) for 30 min at 37 °C in cAMP stimulation buffer (HBSS containing 0.1% BSA (Sigma–Aldrich, Cat#A7906), 5 mM HEPES (ThermoFischer Scientific, Cat#11344-041) and 500 μM IBMX (3-isobutyl-1-methylxanthine; Sigma–Aldrich, Cat#I7018). After stimulation, the buffer was removed and 50 μL of ice cold 100% ethanol added. Cells were dried at room temperature overnight. 50 μL of lysis buffer (HBSS with 0.1% BSA, 0.3% Tween20 (Sigma–Aldrich, Cat#P7949) and 5 mM HEPES) was added to each well and samples mixed on a plate shaker. Cyclic AMP levels were determined using a commercial kit (LANCE cAMP Detection Kit; PerkinElmer, Cat#AD0264) according to the manufacturer's instructions. Briefly, 10 μL of lysate samples or cAMP standards were transferred into a 384 well OptiPlate. In each well, 5 μL of Alexa cAMP antibody mix and 10 μL of detection mix were added sequentially. Plates were read at 615 nm and 665 nm wavelengths on the Envision plate reader (PerkinElmer) after an overnight incubation.

To measure cAMP in L6 cells, the medium of cultured L6-GLUT4-myc cells was changed to a serum-free medium, the day before the experiment. On the day of the experiment, the medium was replaced with a pre-warmed stimulation medium (HBSS containing 5 mM HEPES, 1% BSA and 1 mM of the phosphodiesterase inhibitor IBMX, pH 7.4) and isoprenaline (10 μM) and ATR-127 (10 μM) were added. After 15 min of stimulation, the medium was aspirated, and the cells were stored in 95% ethanol at −20 °C. The ethanol was evaporated and a lysis buffer (ddH_2_O containing 5 mM HEPES, 1% BSA and 0.3% Tween-20, pH 7.4) was added to the cells. The cells were frozen at −80 °C and then stored at −20 °C. On the day of the cAMP measurement, the cells were thawed while nutating at room temperature and the cAMP was measured using the AlphaScreen cAMP assay (PerkinElmer) according to manufacturer's instructions. The plate is analyzed in the Enspire Manager (PerkinElmer).

### 4.6 BRET assays

HEK 293A cells (3.5 × 10^5^ cells/ml) were transfected in suspension with plasmid DNA [adjusted to 1 μg with salmon sperm DNA (Invitrogen)], in complex with PEI (MW 25,000, 3:1 PEI:DNA ratio) and seeded (3.5 × 10^4^ cells/well) in white 96-well plates precoated with PDL. After 48 h the cells were washed with and maintained in HBSS. The cells were incubated with coelenterazine 400a (5 μM) for 5 min at 37 °C and then stimulated with agonist prior to BRET measurement. The plates were read on a Tecan Spark multimode microplate reader, (Tecan; Männedorf, Switzerland) equipped with filters for BRET^2^ (centre wavelength/bandwidth): 400/70 nm (donor) and 515/20 nm (acceptor) for the detection of *R*lucII and rGFP light emissions, respectively.

### 4.7 Receptor internalisation assay

HEK293T cell line was transfected according to the manufacturers’ (Promega, Madison, WI) instructions with SNAP-β_2_AR in pcDNA3 using FuGENE HD transfection reagent. Following transfection, cells with vector incorporated were selected by the addition of 0.5 mg/mL G418 to generate a mixed population stable cell line. Twenty four hours prior to the assay, SNAP-β_2_ HEK293T cells were seeded into poly-d-lysine (10 μg/mL) coated black, μClear-bottomed, 96 well plates (Greiner Bio-One,Stonehouse, UK), at 25,000–30,000 cells per well, and incubated at 37 °C/5% CO_2_ for 24 h prior to the assay. On day of assay, cells were washed in high glucose DMEM/10% FCS and replaced with pre-warmed 0.1 μM membrane-impermeant SNAP-surface AF488 in DMEM/10%FCS and incubated for 30 min at 37 °C/5% CO_2_. Cells were washed with HEPES Balanced Salt Solution (Hepes BSS; 10 mM HEPES, 2 mM sodium pyruvate, 146 mM NaCl, 5 mM KCl, 1 mM MgSO_4_, 1.7 mM CaCl_2_, 1.5 mM NaHCO_3_, 5 mM D-glucose; pH 7.45 with NaOH) with 0.1% BSA (HepesBSS/0.1% BSA) assay buffer and replaced with 50 μg/mL transferrin-AF546 (ThermoFisher, Waltham, MA) and the appropriate concentrations of indicated ligands in HepesBSS/0.1% BSA. Cells were incubated for 1 h at 37 °C/0% CO_2_, before fixation.

After 1 h, cells were fixed with 3% paraformaldehyde, in PBS, for 15 min at room temperature before washing with PBS. Cells were stained with 2 μg/mL Hoechst 33342 nuclear stain (H33342), in PBS, and incubated at room temperature for 15 min, before being replaced with PBS and stored at 4 °C.

Cells were imaged using an ImageXpress Ultra confocal plate reader (Molecular Devices, San Diego, CA, U.S.A.), using 4 sites per well and a Pan Fluor 40x NA 0.6 extra-long working distance objective. Each site was excited using a DAPI, FITC and Texas Red laser filter for H33342, SNAP-AF488 and transferrinAF456 imaging, respectively. Receptor internalisation was analysed using the Translocation Enhanced analysis Module (MetaXpress 5.01, Molecular Devices). The analysis uses an algorithm to identify translocation of AF488-SNAP-β_2_AR (the translocation probe) to transferrin AF456 labelled endosomes (the compartment). The endosomal compartments were defined using based on a set approximate width (3 μm) and both minimum (3 μm^2^) and maximum (100 μm^2^) compartment areas. Compartments were defined for each individual experiment by setting a threshold brightness intensity above background. As a measurement of receptor internalisation, the mean fluorescence intensity of the probe (SNAP-labelled receptor) within the identified transferrin compartments in each image was quantified by the algorithm. Intensity values from 4 sites per well, with assays run in duplicate wells, were normalised between vehicle (0 %) and 10 μM isoprenaline treated wells (100%).

### 4.8 *In vitro* glucose uptake assays

Glucose uptake assays were performed as previously described in L6-GLUT4-myc cells and primary adipocytes [83]. The medium was changed to a serum-free medium the day before the experiment. On the day of the experiment, cells were stimulated for 1 h and 40 min with ATR-127 or positive controls. Positive controls for L6 muscle cells included isoprenaline (10 μM), and norepinephrine (NE) and CL-316,243 (10 μM and 10 μM, respectively) for BAT cells. During the *in vitro* glucose uptake assay in L6 muscle cells, the β_2_-adrenergic receptor antagonist ICI-118,551 (10 μM) was additionally added 30 min before other stimulants. After incubation, the medium was discarded, and cells were washed twice with pre-warmed glucose-free Dulbecco's Modified Eagle Medium (DMEM) and changed to glucose-free DMEM. Compounds were re-administered for 15–20 min at 37 °C and thereafter 2-deoxy-D-[1-3H]-glucose (50 nM) was added to the wells for 10 min. Reactions were terminated by washing with ice-cold glucose free DMEM and the cells were lysed with 0.2 M NaOH, 1 h at 60 °C. The incorporated radio labeled glucose was determined by liquid scintillation counting.

### 4.9 *In vitro* GLUT4 translocation

L6-GLUT4-myc cells were seeded in 8-well μ-slides and differentiated for 6 days prior to the experiment. On the day of the experiment, cells were serum-starved for 3 h and then stimulated for 2 h with ATR-127 (10 μM), vehicle (DMSO) and isoprenaline (10 μM) as positive control. After stimulation, cells were fixed with 4% paraformaldehyde, blocked with glycerine and bovine serum albumin (BSA), and incubated with primary antibody (1:500 rabbit anti-myc) overnight at 4 °C. On the next day, cells were incubated in the dark for 1 h with conjugated Alexa Fluor555 goat anti-rabbit antibody (1:500, Invitrogen). The nuclei were stained for 5 min with Hoechst diluted 1:10000 in PBS. Fluorescence was detected with a fluorescent confocal microscope (Zeiss LSM 780) 20x magnification. Fluorescence intensity was quantified with Fiji (ImageJ).

### 4.10 CalScreener (SymCel)

For heat production measurement in mature adipocytes, we used the calScreener™: a 48-channel isothermal microcalorimeter (Symcel Sverige AB, Spånga, Sweden), with its corresponding 48-well plate (calPlate™) as previously described in M. H. Bokhari et al., 2021 [45]. Each well consists of a screw-capped titanium vial, with sterile single use plastic insert of cell-culture and microscopy grade quality, in which a maximum of 300 μL media can be added (total volume of insert is 600 μL). Data was continuously collected with the corresponding calView™ software (Version 1.0.33.0, 2016, Symcel Sverige AB). For the assays, the apparatus was set and calibrated at 37 °C. For sample loading the mature adipocyte cell suspension was gently transferred into 30-well inserts of the plate in a volume of 200 μL/well. The other 2-well inserts were used as negative controls and loaded with only media to measure and exclude background heating or possible heat reactions of the inserts or working media. The inserts were transferred into the calPlateTM, and the control was added to the wells. As the first viable data are recorded 60 min after the initial sample loading, it is essential that all the measurements are carried out with a controlled starting time. Internal baseline adjustment for every position is essential to achieve correct heat flow measurement data.

### 4.11 Oxygen consumption measurements *in vitro*

Mitochondrial respiration in cultured adipocytes derived from human BAT and WAT were performed essentially as described before [84]. In brief, human primary WAT and BAT adipocyte cells were differentiated in XF96-well plates and oxygen consumption rates were measured using the XF96 extracellular flux analyzer (Seahorse Biosciences, North Billerica, MA). Cells were incubated for 1 h at 37 °C in unbuffered DMEM (2 mM GlutaMAX, 1 mM sodium pyruvate, and 25 mM glucose). Basal oxygen consumption was measured in the presence of 2 μM oligomycin subsequently followed by injection of various concentrations of ATR-127 (n = 8–14 replicates derived from 2 patients).

### 4.12 Animals and ethical approval

C57Bl/6N male mice were purchased from Scanbur (Charles River). Mice had access to food and water ad libitum and were maintained in a 12 h light/dark cycle. During the acute experiment (for more details on the study design see below), mice were group-caged, fed a chow diet (BRAND) and were kept at room temperature (21 °C). During the prolonged diet-induced obesity experiment, mice were on a high-fat (45%) diet (HFD) (Safe diet, U8954 Version 204) for 5 months and were kept at thermoneutrality (30 °C). At the start of the treatment period, mice were single caged. Cages were enriched with wood chips, a cardboard house or a roll, a wooden stick, paper, and a piece of cotton. All procedures were approved by the North Stockholm Ethical Committee for Care and Use of Laboratory Animals.

### 4.13 Study designs

#### 4.13.1 The acute effects of ATR-127 injection on *in vivo* glucose uptake

Ten week old, male C57Bl/6N mice, fasted for 5 h, were anesthetized with pentobarbital (60 mg/kg i.p). They were then injected with either ATR-127 (1 mg/kg), insulin (1 mg/kg) (ActRapid, Novonordisk), clenbuterol (1 mg/kg) (Cat no: C5423, Sigma), CL-316243 (1 mg/kg), or a saline solution. Blood glucose was measured twice, before administration of the drug and before injecting 2-DG, by tail tip cut. Neither ATR-127 nor clenbuterol affected blood glucose of anaesthetized mice when measured 20 min after drugs injection (not shown); thus, glucose uptake was not influenced by different blood glucose levels. After 20 min, 4.81 × 10^6^ Bq/kg of 2-deoxy [3H] glucose (2-DG) (Perkin Elmer, Waltham, MA, USA) was injected intraperitoneally. Animals were killed 1 h later; tissues of interest were dissected out and lysed in 0.2M NaOH. Radioactivity was measured by scintillation counting (Tri-Carb 4810 TR, PerkinElmer).

#### 4.13.2 Effect of prolonged ATR-127 treatment on body weight and whole-body metabolism

At 7-months of age, 16C57Bl/6N male DIO mice upon 5 months on HFD were divided into two homogeneous groups according to their glucose tolerance, body weight and body composition (for a flow-chart of the experimental set-up, please see Suppl. Fig. 2). ATR-127 (5 mg/kg) was dissolved in saline and injected i.p. daily, whereas the control group only received saline. Total duration of the treatment was 21 days. Body weight- and composition were measured every week by EchoMRI-100 (Echo Medical Systems). Food intake was measured every 2–3 days for the duration of the treatment. Glucose tolerance tests (GTTs) were performed before the treatment and after 4- and 11-days of treatment. Blood sampling for serum analysis of insulin and fatty acids was done during last week (day 18). After 3-weeks of treatment, whole-body oxygen consumption was measured by means of metabolic chambers (Promethion, Sable systems). After the treatment period, mice were sacrificed with CO_2_ and subsequent cervical dislocation. Liver, WAT, BAT, skeletal muscle and heart tissues were dissected, weighed, and collected for further analysis.

### 4.14 Glucose tolerance test

Glucose tolerance tests were performed 24 h after the last injection with either ATR-127 or saline. Briefly, mice were fasted for 5 h and intra-peritoneally injected with glucose (2.5 g/kg lean weight). Blood glucose levels were measured prior to glucose administration (fasting t = 0) and 15, 30, 60, 90 and 120 min, post glucose administration. The total AUC was calculated with 0 mmol/L blood glucose as baseline.

### 4.15 Oxygen consumption measurements *in vivo*

After three weeks of treatment, mice were introduced to the metabolic chambers (Promethion, Sable Systems) for acclimatization before administering with either ATR-127 (5 mg/kg) or saline injections. Oxygen consumption was assessed over a 24-hour period, comprising both light phase (7 h–19 h) and dark phase (19 h–7 h). The baseline was determined by averaging measurements taken during a lowest, stable 20-minute period before the administration of ATR-127 or saline. Energy expenditure during light phase is determined by averaging measurements taken during 7 h–19 h.

### 4.16 Free fatty acids, cholesterol, and insulin measurements from serum

Serum levels of free fatty acids, HDLc, LDLc, total cholesterol and insulin levels were measured using commercially available kits. Murine Free Fatty Acid Assay Kit (Abcam, Catalog no: 65341); Total cholesterol, HDLc and LDLc kit (Abcam, Catalog no: 65390) and Insulin ELISA kit (CrystalChem, Catalog no: 90080) according to the manufacturer's instructions.

### 4.17 Hepatic lipids droplets imaging

Fluorescence dye BODIPY 495/503 (ThermoScientific, Catalog no: D3922) was applied on 10 μm frozen liver sections for 10 min at room temperature. After one wash in PBS, slides were assembled with mounting gel and images were obtained using LSM-780 fluorescence confocal microscope.

### 4.18 Real time PCR

Total RNA was isolated from mouse liver, BAT tissue samples and primary human BAT cells with TRI Reagent (Sigma–Aldrich, Catlog no: T9424) following the manufacturer's instructions. For real-time PCR of mouse liver and BAT tissues, 500 ng total RNA was reverse transcribed using random hexamer primers, deoxynucleoside triphosphates, MultiScribe reverse transcriptase, and RNase inhibitor (Applied Biosystems, Foster City, CA). cDNA (5 ng) samples were run in duplicates. Thermal cycling conditions were 2 min at 50 °C, 10 min at 95 °C, and 40 cycles of 15 s at 95 °C and 1 min at 60 °C, followed by the melting curve analysis on a Bio-Rad CFX Connect Real-Time system. Transcription factor IIB (TFIIB) was used as housekeeping genes for quantity normalization.

From primary human BAT cells, total RNA (20 ng) was reverse transcribed using the High-Capacity cDNA Reverse Transcription Kit (Applied Biosystems). cDNA samples were run in triplicates and qPCR was performed using ViiA7 Sequence Detection system (Applied Biosystems, Foster City, CA, USA) according to the manufacturer's protocol using the PowerUp SYBR Green Master Mix (Thermo Fisher). Prolyl peptidyl isomerase A (PPIA) was used as housekeeping gene for quantity normalization. Sequences of the oligonucleotide primers are listed in Suppl table 1.

### 4.19 Statistical analysis

Data are expressed as mean ± SEM and were analyzed by means of Prism 9 software (GraphPad Software, San Diego, CA). Differences between two or multiple groups were analyzed by means of the Student's *t* test or one-way ANOVA followed by the Dunnett's or Sidak's multiple comparison tests. A significant difference was considered if p < 0.05. The annotations used are ∗p < 0.05, ∗∗p < 0.01, ∗∗∗p < 0.001. For BRET analysis, curve fitting was performed by three parameter nonlinear regression using GraphPad Prism 9.4.0 (GraphPad Software, San Diego, CA, USA). Analyses were performed using the extra sum-of-squares F test, for testing the statistical difference between the top and bottom parameters of the non-linear regression. Best fit values were excluded if EC_50_ was outside of the concentration range tested or if the span was negative. The Benjamini-Hochberg correction was applied to account for multiple comparisons. (P < 0.0043).

Contractile force is represented as a % of the inotropic effect of the full agonist (−)-isoprenaline and all calculations to determine % of (−)-isoprenaline were performed in Excel Version 2016. Determination of logEC_50_ values were analyzed with GraphPad Prism Version 8.3.1 using non-linear curve fitting with the equation: Y=Bottom + (Top-Bottom)/(1 + 10ˆ((logEC50-X)∗HillSlope).

## Funding

T.B is supported by open access funding provided by Stockholm University. The study was supported by Vetenskapsrådet-Medicin (VR-M) from the Swedish Research Council, Eurostar project Synstar and Diabetes Wellness Sverige. S.C.W. is supported by the Swedish Society for Medical Research (P18-0098; PD20-0153). V.M.L. is supported by the Swedish Research Council (grant agreement numbers 2019-01837 and 2021-02801), by the EU/EFPIA/OICR/McGill/KTH/Diamond Innovative Medicines Initiative 2 Joint Undertaking (EUbOPEN grant number 875510), by the Swedish Strategic Research Programme in Diabetes (SFO Diabetes) and Stem Cells and Regenerative Medicine (SFO StratRegen), by the European Union's Horizon 2020 research and innovation program U-PGx (grant agreement number 668353), and by the Robert Bosch Foundation, Stuttgart, Germany.

## CRediT authorship contribution statement

**Emanuela Talamonti:** Writing – original draft, Formal analysis, Data curation, Conceptualization. **Jelena Davegardh:** Writing – original draft, Formal analysis, Data curation, Conceptualization. **Anastasia Kalinovich:** Formal analysis, Data curation, Conceptualization. **Sten M.M. van Beek:** Writing – original draft. **Nodi Dehvari:** Conceptualization, Data curation, Supervision. **Carina Halleskog:** Supervision, Formal analysis, Data curation. **Hamza M. Bokhari:** Formal analysis, Writing – review & editing. **Dana S. Hutchinson:** Supervision, Data curation, Formal analysis. **Seungmin Ham:** Formal analysis, Data curation. **Laura J. Humphrys:** Formal analysis, Data curation. **Nicola C. Dijon:** Formal analysis, Data curation. **Aikaterini Motso:** Data curation, Formal analysis. **Anna Sandstrom:** Data curation, Formal analysis. **Evelyn Zacharewicz:** Data curation, Formal analysis. **Ilga Mutule:** Formal analysis, Supervision. **Edgars Suna:** Formal analysis, Investigation, Supervision. **Jana Spura:** Data curation, Formal analysis. **Karolina Ditrychova:** Data curation, Formal analysis. **Leigh A. Stoddart:** Data curation, Formal analysis, Supervision. **Nicholas D. Holliday:** Formal analysis, Supervision. **Shane C. Wright:** Data curation, Formal analysis, Supervision, Writing – review & editing. **Volker M. Lauschke:** Formal analysis, Supervision. **Soren Nielsen:** Formal analysis, Supervision. **Camilla Scheele:** Formal analysis, Supervision. **Elizabeth Cheesman:** Data curation, Formal analysis. **Joris Hoeks:** Supervision, Formal analysis. **Peter Molenaar:** Supervision, Formal analysis. **Roger J. Summers:** Writing – review & editing, Supervision, Formal analysis. **Benjamin Pelcman:** Supervision, Formal analysis, Data curation, Conceptualization. **Gopala K. Yakala:** Writing – original draft, Supervision, Formal analysis, Data curation. **Tore Bengtsson:** Writing – review & editing, Supervision, Funding acquisition, Conceptualization.

## Declaration of competing interest

The authors declare the following financial interests/personal relationships which may be considered as potential competing interests: TB, BP and ND own stocks in Atrogi AB. ET, AK, ND, SvB, CH, MHB, AS, BP and GY are employed by Atrogi AB. VML is co-founder, CEO and shareholder of HepaPredict AB.

## Data Availability

Data will be made available on request.
