# Supplementary material for: The novel adrenergic agonist ATR-127 targets skeletal muscle and brown adipose tissue to tackle diabesity and steatohepatitis
Source: Mol Metab. 2024 May 17;85:101931. doi: 10.1016/j.molmet.2024.101931 (PMC11258667; doi:10.1016/j.molmet.2024.101931)
Supplement: Multimedia component 1 [file mmc1.docx]

**The novel adrenergic agonist ATR-127 targets skeletal muscle and brown adipose tissue to tackle diabesity and steatohepatitis**

Emanuela Talamonti^1^#, Jelena Davegardh^2^#, Anastasia Kalinovich^1^, Sten M M van Beek^1^, Nodi Dehvari^1^, Carina Halleskog^1^, Hamza M Bokhari^1^, Dana S Hutchinson^3^, Seungmin Ham^3^, Laura J Humphrys^4^, Nicola C Dijon^4^, Aikaterini Motso^2,5^, Anna Sandstrom^1^, Evelyn Zacharewicz^6^, Ilga Mutule^7^, Edgars Suna^7^, Jana Spura^7^, Karolina Ditrychova^11^, Leigh A Stoddart^8^, Nicholas D Holliday^4,8^, Shane C Wright^5^, Volker M Lauschke^5,9,10^, Soren Nielsen^11^, Camilla Scheele^11^, Elizabeth Cheesman^12^, Joris Hoeks^6^, Peter Molenaar^12,13^, Roger J Summers^3^, Benjamin Pelcman^1^, Gopala K Yakala^1*^, Tore Bengtsson^1,2*^

# Authors contributed equally

*Authors contributed equally

1. Atrogi AB, Tomtebodavagen 6, Solna, Stockholm, Sweden.

2. Department of Molecular Biosciences, The Wenner-Gren Institute, Stockholm University, Stockholm, Sweden.

3. Drug Discovery Biology, Monash Institute of Pharmaceutical Sciences, Monash University, Parkville, Victoria, Australia.

4. School of Life Sciences, The Medical School, Queen’s Medical Centre, University of Nottingham, Nottingham, UK.

5. Karolinska Institutet, Department of Physiology and Pharmacology, Stockholm, Sweden.

6. Department of Nutrition and Movement Sciences, NUTRIM School of Nutrition and Translational Research in Metabolism, Maastricht University Medical Center, Maastricht, the Netherlands.

7. Latvian Institute of Organic Synthesis, Riga, Latvia.

8. Excellerate Bioscience, The Triangle, NG2 Business Park, Nottingham, UK.

9. Dr. Margarete Fischer-Bosch Institute of Clinical Pharmacology, Stuttgart, Germany

10. Tübingen University, Tübingen, German

11. Novo Nordisk Foundation Center for Basic Metabolic Research, Faculty of Health and Medical Sciences, University of Copenhagen, Copenhagen, Denmark; The Centre of Inflammation and Metabolism and Centre for Physical Activity Research, Righospitalet, University Hospital of Copenhagen, Copenhagen, Denmark.

12. Cardio-Vascular Molecular & Therapeutics Translational Research Group, Northside Clinical School of Medicine, Faculty of Medicine, University of Queensland, Brisbane, Queensland, Australia.

13. Queensland University of Technology (QUT), School of Biomedical Sciences, Institute of Health and Biomedical Innovation, 60 Musk Avenue, Kelvin Grove, Queensland, Australia.

Synthesis of ATR-127


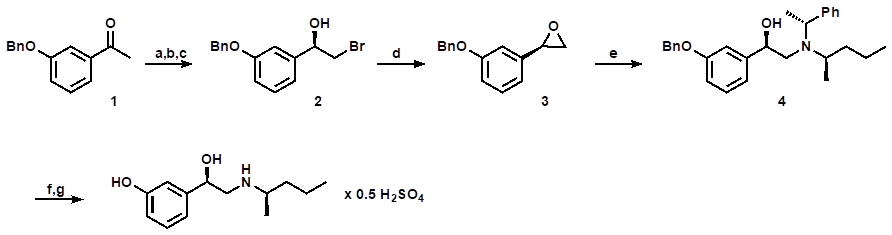


Steps a and b


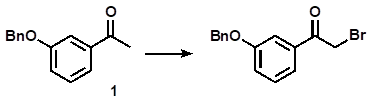


3-Benzyloxyphenacyl bromide.

A solution of Br_2_ (4.5 mL, 88.4 mmol) in CHCl_3_ (150 mL) was added over ≈4 h to a solution of 3‑benzyloxyacetophenone (**1**) (10.0 g, 44.2 mmol) in CHCl_3_ (200 mL) at reflux and heating was continued until complete conversion (≈1h). The solution was cooled to rt and concentrated, and the crude mixture of mono- and di-brominated products was dissolved in THF (70 mL) and cooled to 0° C. A solution of diethylphosphite (5.7 mL, 44.2 mmol) and Et_3_N (6.2 mL, 44.2 mmol) in THF (70 mL) was slowly added to the mixture of products over 30 min. The mixture was allowed to warm to rt and was stirred for 30 min and then poured into ice water (800 mL) and stirred for 2 h. The precipitate was filtered off, washed with water and air-dried. The material was purified by column chromatography (silica gel, PE:EtOAc, 10:1) and crystallized (PE:Et_2_O, 10:1), to give the product as an off-white solid (8.6 g, 63%) [1].

R_f_=0.28, (PE:EtOAc, 10:1); ^1^H NMR (400 MHz, CDCl_3_ δ 7.64-7.54 (m, 2H), 7.49 – 7.30 (m, 6H), 7.23 (ddd, J = 8.3, 2.6, 1.0 Hz, 1H), 5.12 (s, 2H), 4.43 (s, 2H); ^13^C NMR (101 MHz, CDCl_3_) δ 191.18, 159.27, 136.44, 135.43, 130.05, 128.84, 128.37, 127.72, 121.86, 121.36, 114.39, 70.43, 31.11.

Step c


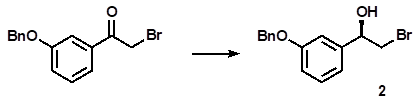


(R)-1-(3-(Benzyloxy)phenyl)-2-bromoethan-1-ol (**2**).

Borane dimethylsulfide complex (0.5 mL, 5.2 mmol) was added dropwise to a stirred solution of (*R*)-2-methyl-CBS-oxazaborolidine (1 M in toluene, 0.66 mL, 0.66 mmol) in THF (1.5 mL) at 0 °C under Ar. After 30 min stirring, a solution of 3-benzyloxyphenacyl bromide (2.0 g, 6.6 mmol) in THF (3.7 mL) was added using a syringe pump (0.09 mL/min) while keeping the temperature at 4–7 °C. The mixture was stirred at rt for 1.5 h and MeOH (5 mL) was added at 0 °C. The mixture was stirred for 30 min and concentrated and the residue was purified using column chromatography (silica gel, PE:EtOAc, 20:1), giving compound **2** as colorless oil (1.83 g, 91%).

R_f_=0.30, PE:Et_2_O, 2:1; [α]^D^_20_ =-0.30 (c=1.0, CHCl_3_); ^1^H NMR (400 MHz, CDCl_3_) δ 7.46 – 7.27 (m, 5H), 7.06 – 7.03 (m, 1H), 6.99 – 6.92 (m, 2H), 5.08 (s, 2H), 4.90 (dt, J = 9.0, 3.2 Hz, 1H), 3.64 (dd, J = 10.5, 3.3 Hz, 1H), 3.53 (dd, J = 10.5, 9.0 Hz, 1H), 2.61 (d, J = 3.3 Hz, 1H); ^13^C NMR (101 MHz, CDCl_3_) δ 159.09, 141.92, 136.77, 129.79, 128.61, 128.04, 127.52, 118.53, 114.83, 112.48, 73.70, 70.06, 40.19;

Chiral HPLC analysis: column Daicel CHIRALPAK ID (4.6 mm x 250 mm, 5 μm), eluent n‑heptane:iPrOH (96:4), flow rate 1.0 mL/min, detection wavelength 260 nm, t_R_ (S) = 19.29 min (0.8%), t_R_ (R) = 24.99 min (99.2%), ee = 98%.

Step d


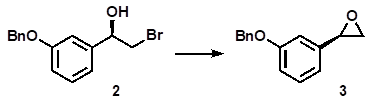


(*R*)-2-(3-(Benzyloxy)phenyl)oxirane (**3**).

K_2_CO_3_ (1.23 g, 8.9 mmol) was added to a solution of (*R*)-1-(3-(benzyloxy)phenyl)-2-bromoethan-1-ol **2** (1.83 g, 5.6 mmol) in MeOH (36 mL) and the mixture was stirred at rt for 1 h. The mixture was concentrated and CH_2_Cl_2_ (20 mL) was added to the residue and the solids were filtered off and washed with CH_2_Cl_2_. The solvents of the filtrate were removed in vacuo to give compound **3** as colorless oil (1.28 g, 95%).

R_f_=0.30 (PE:EtOAc, 10:1, KMnO_4_ stain); [α]^D^_20_ =-4.2 (c=1.0, CHCl_3_); ^1^H NMR (400 MHz, CDCl_3_) δ 7.50 – 7.20 (m, 6H), 6.94 – 6.88 (m, 3H), 5.07 (s, 2H), 3.84 (dd, J = 4.0, 2.6 Hz, 1H), 3.13 (dd, J = 5.6, 4.1 Hz, 1H), 2.77 (dd, J = 5.6, 2.6 Hz, 1H); ^13^C NMR (101 MHz, CDCl_3_) δ 159.27, 139.50, 136.99, 129.76, 128.74, 128.14, 127.64, 118.41, 114.90, 111.70, 70.15, 52.44, 51.33.

Step e


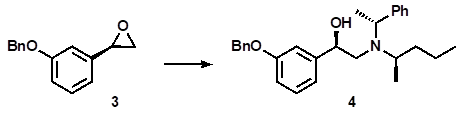


(*R*)-1-(3-(Benzyloxy)phenyl)-2-(((*R*)-pentan-2-yl)((R)-1-phenylethyl)amino)ethan-1-ol (**4**).

A mixture of **3** (1.28 g, 5.64 mmol), (*R*)-*N*-((*R*)-1-phenylethyl)pentan-2-amine (0.54 g, 2.82 mM) and iPrOH (0.43 mL, 5.65 mmol) was heated for 88 h at 140 °C in a pressure vial. The mixture was concentrated under reduced pressure and the residue was purified by column chromatography (silica gel, PE:Et_2_O (2-10%)) to give compound **4** as yellow oil (0.85 g, 72%).

R_f_ = 0.20 (10:1, PE/EtOAc, KMnO_4_ stain); [α]^D^_20_ =-79.2 (c=0.79, CHCl_3_); ^1^H NMR (400 MHz, CDCl_3_) δ 7.49 – 7.19 (m, 11H), 6.98-6.91 (m, 1H), 6.91 – 6.82 (m, 2H), 5.06 (s, 2H), 4.33 (dd, J = 10.6, 3.3 Hz, 1H), 4.08 (q, J = 6.8 Hz, 1H), 2.92 (h, J = 6.1 Hz, 1H), 2.75 (dd, J = 13.5, 3.4 Hz, 1H), 2.44 (dd, J = 13.3, 10.9 Hz, 1H), 2.3-1.3 (br s, 1H, overlapping), 1.44 (d, J = 6.8 Hz, 3H), 1.42-1.34 (m, 1H), 1.31 – 1.07 (m, 3H), 1.00 (d, J = 6.6 Hz, 3H), 0.77 (t, J = 7.0 Hz, 3H); ^13^C NMR (101 MHz, CDCl_3_) δ 159.02, 144.84, 144.48, 137.24, 129.38, 128.69, 128.47, 128.10, 128.05, 127.69, 127.24, 118.61, 113.77, 112.37, 70.09, 69.26, 57.49, 54.63, 51.87, 38.72, 20.21, 16.92, 16.32, 14.13.

Step f


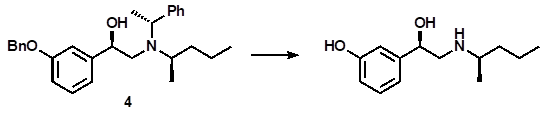


3-((*R*)-1-Hydroxy-2-(((*R*)-pentan-2-yl)amino)ethyl)phenol.

Et_3_SiH (3.2 mL, 20 mmol) was added dropwise to a stirred suspension of **4** (830 mg, 2.0 mmol) and 10% Pd(C) (423 mg, 0.4 mmol) in MeOH (7 mL) at rt. The reaction mixture was stirred at rt for 1 h and filtered through Celite which was washed with MeOH. The combined filtrates were concentrated in vacuo and the residue purified on a short silica gel column (PE:Et_2_O, 2:1), followed by CH_2_Cl_2_:MeOH (10:1) to give the product as light-yellow oil (330 mg, 74%).

Step g


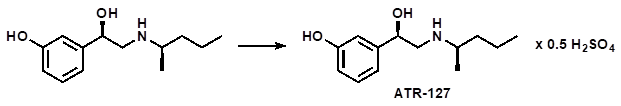


3-((*R*)-1-Hydroxy-2-(((*R*)-pentan-2-yl)amino)ethyl)phenol hemisulfate (**ATR-127**).

H_2_SO_4_ (0.1 M in H_2_O, 7.24 mL, 0.724 mmol) was added to a solution of 3-((*R*)-1-hydroxy-2-(((*R*)-pentan-2-yl)amino)ethyl)phenol (323 mg, 1.45 mmol) in H_2_O (16 mL) and the mixture was stirred at rt for 1 h and concentrated to dryness. Toluene was added to the residue and the mixture was concentrated in vacuo. This procedure was repeated two more times. The residue was suspended in Et_2_O and filtered and the solids dried in vacuo over P_2_O_5_ to afford ATR-127 as an off-white solid (325 mg, 82%).

[α]_D_^20^ -12.6 (*c* 1.0, water); HRMS (ESI+) m/z [M+H]^+^ calcd for C_13_H_22_NO_2_, 224.1651; found 224.1657; ^1^H NMR (400 MHz, D_2_O) δ 7.38-7.30 (m, 1H), 7.07 – 6.82 (m, 3H), 4.98 (dd, J = 9.0, 3.4 Hz, 1H), 3.46 – 3.13 (m, 3H), 1.82 – 1.24 (m, 4H), 1.31 (d, J = 6.6 Hz, 3H, overlapping), 0.92 (t, J = 7.2 Hz, 3H); ^13^C NMR (101 MHz, D_2_O) δ 155.86, 141.66, 130.41, 117.89, 115.53, 112.73, 68.76, 54.48, 49.78, 34.40, 18.11, 15.12, 12.87.

Synthesis of the chiral amine used in Step e following a literature procedure for asymmetric reductive amination of prochiral ketones [2].


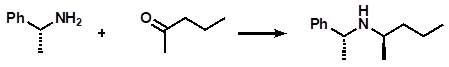


(*R*)-N-((*R*)-1-Phenylethyl)pentan-2-amine hydrobromide.

A solution of (*R*)-2-phenylethylamine (7.74 g, 63.9 mmol, 99% ee) in CH_2_Cl_2_ (20 mL) was added to a solution of 2-pentanone (5.0 g, 58.1 mmol) in CH_2_Cl_2_ (40 mL) at rt followed by Ti(i-PrO)_3_ (20.6 g, 72.6 mmol) and the solution was stirred at rt for 30 min. Raney-Nickel (wet, 70–100 wt%, 16 g) was pre-triturated with EtOH (3x15 mL) and CH_2_Cl_2_ (3x15 mL). This material was suspended in CH_2_Cl_2_ and transferred to a hydrogenation vessel. The imine reaction mixture was added and the mixture was hydrogenated at 20 atm at rt for 16 h. NaOH (1 M, 100 mL) was added and the mixture was stirred at rt for 1 h and filtered through Celite which was washed with CH_2_Cl_2_. The combined filtrates were concentrated to remove the low boiling organics and the remaining aqueous solution was extracted with CH_2_Cl_2_ (3x100 mL). The combined extracts were dried (Na_2_SO_4_), filtered, concentrated and dissolved in CH_2_Cl_2_. Silica gel (200 mL) was added and the solvents were removed in vacuo. A sintered glass filtering funnel (d=10 cm, h=10 cm) was charged with silica gel (100 mL) and the crude product absorbed on silica gel was added and covered with a filter paper. The solids were washed with PE:EtOAc (2:1) (3X300 mL) and the combined filtrates were concentrated to give the product as a colorless oil (9.1 g, 82%). The diastereomeric ratio was determined by ^1^H NMR to be 83:17, (*R,R*): (*R,S*). The material was dissolved in EtOH (68 mL), cooled in ice bath and HBr (33% in AcOH, 18.2 mL, 319.4 mmol) was added dropwise. The mixture was stirred at 0 °C for 1 h and the solid was filtered off and washed with cold EtOH and air-dried for 2 h and over NaOH overnight. The diastereomeric ratio was determined by ^1^H NMR to be 95:5 (R,R):(R,S). The material was dissolved in EtOH:Et_2_O (1:1), 400 mL at 45 °C. While stirring, the solution was allowed to cool down to 35 °C and crystals of pure diastereomer were added. The suspension was stirred at rt overnight and in an ice bath for 1 h and filtered. The solid was washed with cold EtOH:Et_2_O (1:1) and dried in air to give the product as a colorless solid as a single diastereomer (6.88 g, 53%).

[α]^D^_20_ =61.7 (c=4.8, EtOH); ^1^H NMR (400 MHz, CD_3_OD) δ 7.58 – 7.42 (m, 5H), 4.57 (q, J = 6.8 Hz, 1H), 3.03 (dqd, J = 10.1, 6.6, 3.5 Hz, 1H), 1.89-1.79 (m, 1H), 1.67 (d, J = 6.8 Hz, 3H), 1.56 – 1.37 (m, 2H), 1.28 (d, J = 6.6 Hz, 3H), 1.31-1.19 (m, 1H, overlapping), 0.92 (t, J = 7.3 Hz, 3H); ^13^C NMR (101 MHz, CD_3_OD) δ 137.91, 130.68, 130.59, 128.59, 56.69, 53.43, 35.40, 19.96, 19.64, 16.93, 13.94.

(*R*)-N-((*R*)-1-Phenylethyl)pentan-2-amine.

Prior to use, the (*R*)-N-((*R*)-1-phenylethyl)pentan-2-amine hydrobromide is suspended in CH_2_Cl_2_ and treated with concentrated aqueous NaHCO_3_. The organic layer is collected, dried over Na_2_SO_4_ and concentrated.

[1] Hernandez, A., Rapoport, H., *J. Org. Chem.* **1994**, 59, 5, 1058–1066.

[2] Nugent, T. C., Ghosh, A. K., Wakchaure, V. N., Mohantya, R. R., *Adv. Synth. Catal.* **2006**, 348, 1289 – 1299.

**Suppl. Figure 1.** Concentration response curves of isoprenaline and ATR-127 across 15 pathways monitoring biosensor recruitment to the plasma membrane using rGFP-CAAX. Data are represented as the mean ± SEM (*n*=3-4 biologically independent experiments).

**
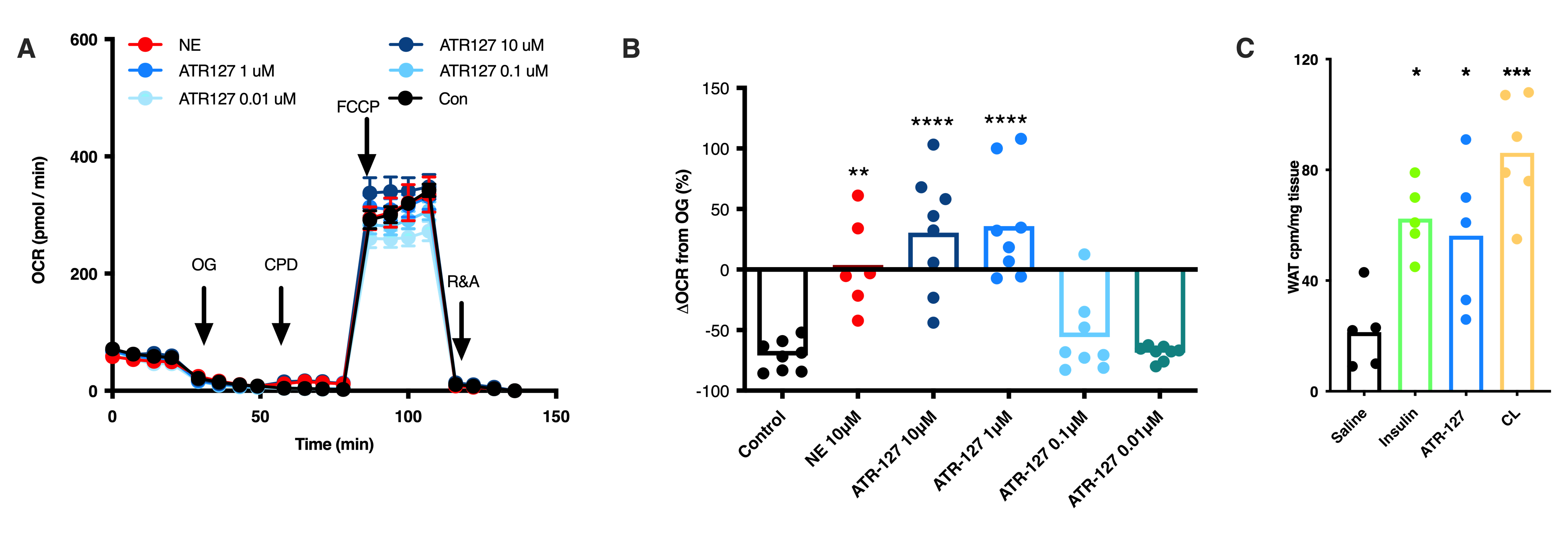
**

**Suppl Figure 2. Effect of ATR-127 on white adipose tissue.** (A) *In vitro* oxygen consumption rate of human white adipocytes stimulated with norepinephrine or ATR-127 at different concentrations. (B) Quantification of oxygen consumption rates calculated as increase over OG % response. (C) *in vivo* glucose uptake into white adipose tissue of C57Bl/N6 mice upon acute injection with ATR-127 (5mg/Kg). Data were analysed by Student t test. * p < 0.05, ** p < 0.01, *** p < 0.001. (h)WAT = (human) white adipose tissue, OCR = oxygen consumption rate, OG = Oligomycin, CPD = Compound (ATR-127 or NE), NE = norepinephrine, CL = CL316.243.

**
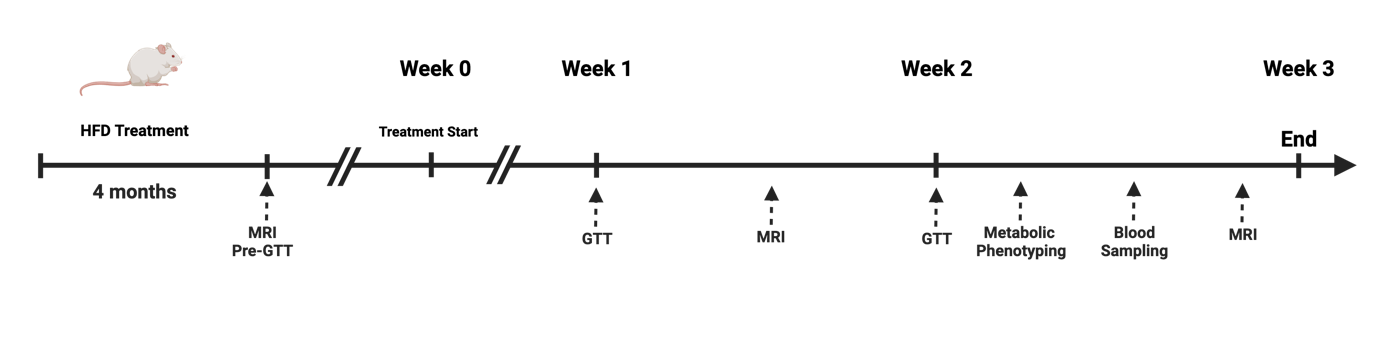
 Suppl. Figure 3. Schematic representation of the experimental design.** Diet-induced obesity was developed in C57Bl/6N mice maintained at 30°C and on HFD for 4 months; DIO mice were treated daily with 5 mg/kg ATR-127 for 3 weeks; MRI and GTT were performed every week; the last week of the treatment mice were placed in metabolic chambers to measure oxygen consumption and blood was collected to measure lipids and insulin content in plasma. Finally, mice were sacrificed, and organs were collected for furth analysis. Created with Biorender (Biorender.com).

**
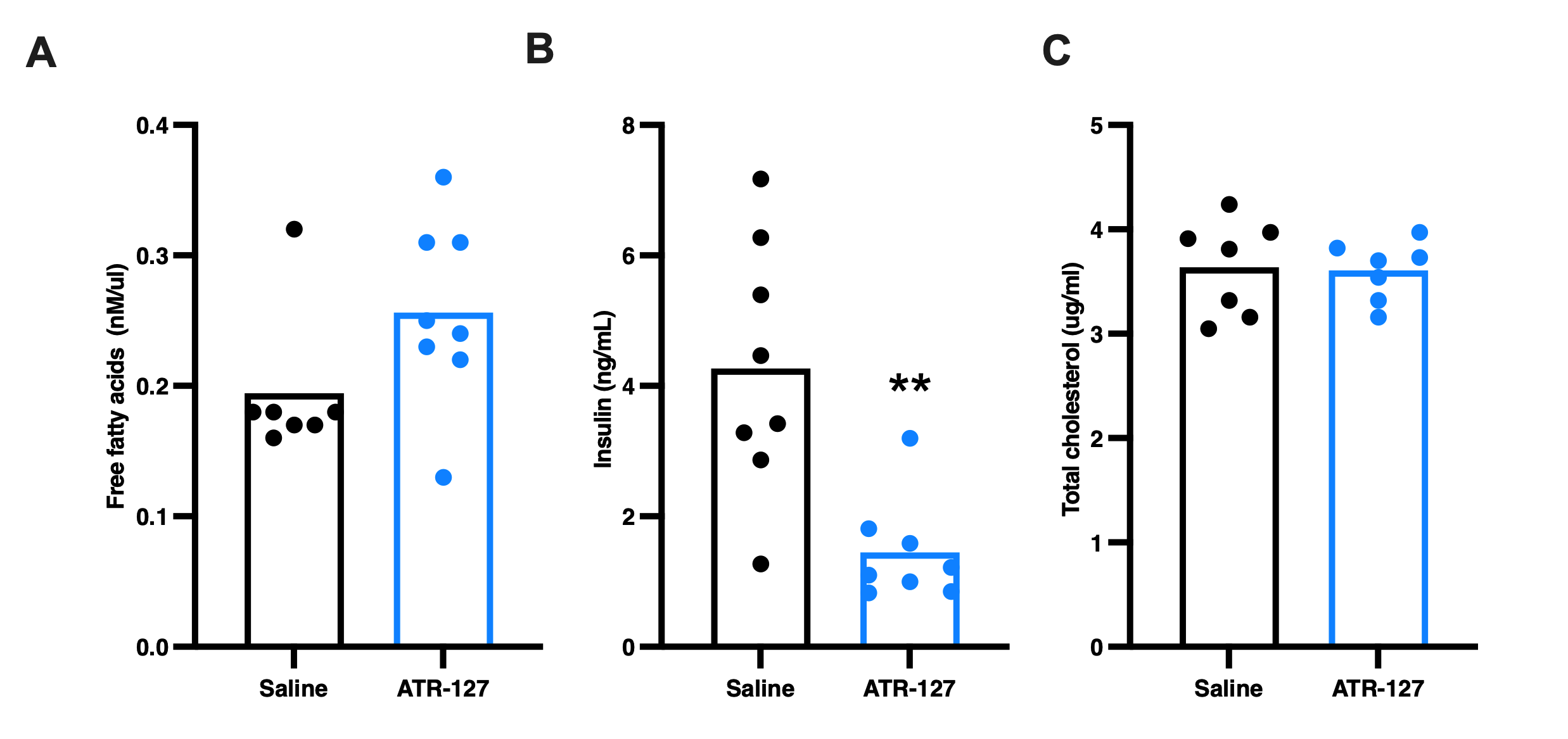
**

**Suppl Figure 4. Effects of prolonged treatment with ATR-127 on plasma lipids and insulin.** Blood was collected after 18 days treatment and mice were fasted in the morning for 5 h prior to blood collection. (A) Chronic effect of ATR-127 on insulin (A), free fatty acids (B) and cholesterol (C) in plasma. Data were analysed by Student t test. A significant difference was considered at ** p < 0.01.

**
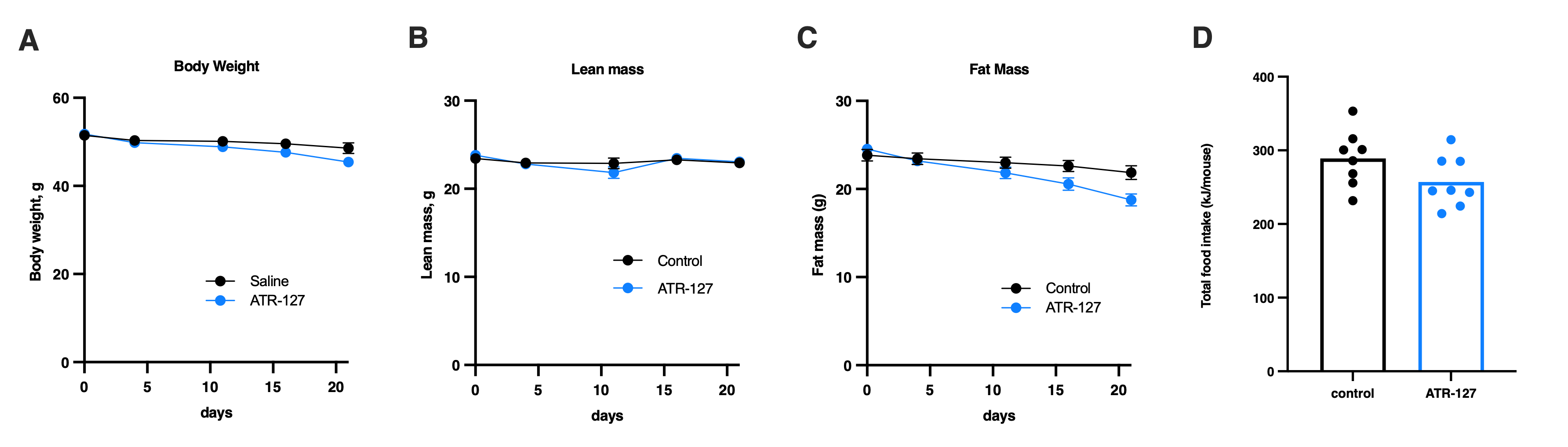
**

**Suppl Figure 5. Effects of prolonged treatment with ATR-127 on body weight, food intake, fat mass and lean mass.** Diet-induced obesity was developed in C57Bl/6N mice maintained at 30 °C and on HFD for 4 months; DIO mice were treated daily with 5 mg/kg ATR-127 for 3 weeks; n =8. (A) Body weight, (B) Food intake (C) Fat mass, (D) Lean mass. The data were analysed by two-way ANOVA with Sidak’s multiple comparison test or Student t test.

**
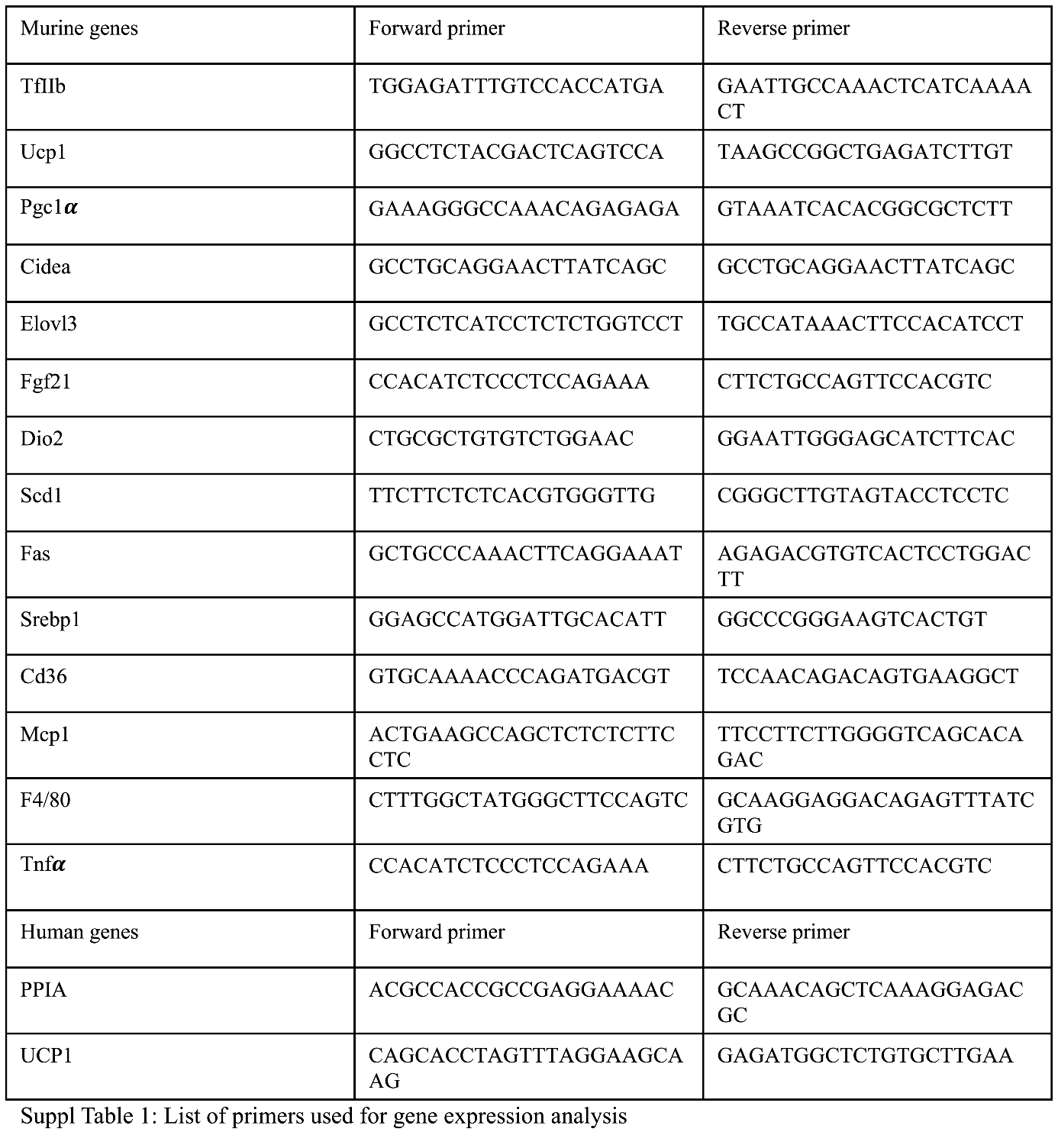
**

**Suppl Figure 6. Effects of prolonged treatment with ATR-127 on oxygen consumption and energy expenditure in vivo.** Diet-induced obesity was developed in C57Bl/6N mice maintained at 30 °C and on HFD for 4 months; DIO mice were treated daily with 5 mg/kg ATR-127 for 3 weeks; n =8. A. 24-hour oxygen consumption comprising light phase and dark phase. The data were analysed using two-way ANOVA. A significant difference was considered at ****p<0.0001.
